# Lineage-independent retrotransposition of UTP14 associated with male fertility has occurred multiple times throughout mammalian evolution

**DOI:** 10.1098/rsos.171049

**Published:** 2017-12-20

**Authors:** Jan Rohozinski

**Affiliations:** 1 Institute for Regenerative Medicine, Wake Forest School of Medicine, 391 Technology Way, Winston-Salem, NC 27101, USA; 2 Division of Gynecologic Oncology, Department of Obstetrics and Gynecology, Center for Reproductive Medicine and Dan L. Duncan Cancer Center, Baylor College of Medicine, Houston, TX 77030, USA

**Keywords:** mammalian evolution, UTP14 retrogenes, convergent recruitment, male reproductive advantage, primate diversity, spermatogenesis

## Abstract

In mammals, gamete production is essential for reproductive success. This is particularly true for males where large quantities of sperm are produced to fertilize a limited number of eggs released by the female. Because of this, new genes associated with increased spermatogenic efficiency have been accumulating throughout the evolution of therian mammals. Many of these new genes are testis-specific retrotransposed copies of housekeeping genes located on the X chromosome. Of particular interest are retrotransposed copies of UTP14 that are present in many distantly related eutherian mammals. Analysis of genomic data available in ENSEMBL indicates that these UTP14 retrogenes have arisen independently in the various eutherian clades. They represent an interesting aspect of evolution whereby new homologues of UTP14 have become independently fixed in multiple mammalian lineages due to the reproductive advantage that may be conferred to males. Surprisingly, these genes may also be lost, even after being present within a lineage for millions of years. This phenomenon may potentially be used to delineate evolutionary trees in closely related groups of mammals, particularly in the case of South American primates. Studying these retrogenes will yield new insights into the evolutionary history of male gamete production and the phylogeny of eutherian mammals.

## 1. Introduction

There are two essential elements for mammalian evolution to proceed. The first is the requirement that living individuals fit within their environment, where they are subject to direct selective pressures, and survive long enough to mate and raise offspring. The second is a requirement that living individuals produce viable gametes so that genetic material can be transmitted to subsequent generations. In mammals, this second requirement is particularly important for male members of a population, where large numbers of sperm are produced to fertilize a limited number of eggs produced by the female. Efficient sperm production may also confer a significant advantage in situations where mating strategies involve sperm competition [1]. Any genomic changes that increase the efficiency of sperm production can have a disproportionate effect on a male's reproductive success. Therefore, it follows that new genes which specifically enhance spermatogenesis can be rapidly fixed within mammalian genomes.

An interesting example of such gene acquisition is the presence of a novel family of retrogenes, the spermatogenesis-associated retrogenes, found within therian genomes that display a testis-specific pattern of expression. Retrotransposition is a process by which duplicate copies of genes are generated via the insertion of reverse-transcribed DNA copies of messenger RNAs into genomic DNA [2]. Gene duplication allows for the relaxation of any functional restraints which the parental gene was subject to. In turn this allows for functional divergence, or specialization, that was not previously possible and subsequently drives complexity within biological systems [2–4]. The presence of spermatogenesis-associated family retrogenes within therian genomes is well documented [3,5]. Their appearance is directly linked to the evolution of distinct X and Y sex chromosomes in therian mammals and the subsequent acquisition of meiotic sex chromosome inactivation during spermatogenesis [6].

The most ancient retrotransposition event that created a spermatogenesis-associated retrogene appears to be that which gave rise to the retrogene PGK2 by duplication of the X-linked gene, PGK1 [7]. A copy of this retrogene is present in all therian genomic sequences currently available. In eutherian mammals, the PGK2 gene is the result of a single retrotransposition event that placed a copy of PGK1 adjacent to, or within, a cluster of CRISP genes (CRISP1, 2 and 3), and this arrangement has been preserved in all eutherian genomes. Since this original retrotransposition event, new spermatogenesis-associated retrogenes have been accumulating within eutherian genomes in a time-dependent pattern [7]. These retrotransposition events thus follow the evolutionary history of eutherian mammals and could potentially be useful in defining evolutionary pathways and taxonomic relationships.

Of particular interest is the evolutionary history of the retrotransposition events involving housekeeping gene UTP14 (U3 small nucleolar RNA-associated protein 14 homologue (yeast)). UTP14 is a highly conserved protein present in all eukaryotes that was first identified in yeast [8] and is located on the X chromosome in all therian mammals. It would appear that this X chromosome localization of UTP14 occurred early in the evolution of the sex-determining chromosomes, soon after divergence from monotremes. The UTP14 gene consists of multiple introns and exons that are processed into a mature mRNA that encodes the functional peptide. This gene has retrotranspositioned multiple times throughout mammalian evolution and, although absent in metatherian mammals, copies of UTP14 retrogenes can be found in all major eutherian branches. These retrogenes can be observed in extant species where they may be present as single or multiple copies with distinct genomic insertion points. In many cases, these retrogenes are present as remnants which do not code for intact peptides. In other cases, there appears to be no identifiable trace of any UTP14 retrogene within a species genome, suggesting that either retrotransposition of UTP14 has never occurred within a lineage or alternatively any UTP14 retrogenes once present within a lineage have been lost. Direct evidence for the biological function of UTP14 retrogenes has been established in mice and humans. In mice, loss of UTP14 retrogene function is associated with a complete loss of spermatogenesis that renders males infertile without any other phenotypic impact in either males or females [9,10]. In the case of humans, mutations within the UTP14 retrogenes are linked to azoospermia that is associated with male infertility [11]. In this paper, the evolutionary history of UTP14 retrotransposition is explored. Copies of this retrogene present within the various phylogenetic groups have been identified, documented and discussed in terms of their evolutionary significance.

## 2. Material and methods

All genomic data were obtained from the ENSEMBL genome database [12] (release 85—July 2016). UTP14 retrogenes were identified by using the ENSEMBL BLAST search facility with Human UTP14A as the reference sequence. Because the coding sequence of UTP14 retrogenes is intronless, they could be readily identified in all genomes and were translated into a peptide sequence using EMBOSS Sixpack (http://www.ebi.ac.uk/Tools/st/emboss_sixpack/). Multi-sequence alignments were done using either Clustal Omega or T-Coffee, which are available on the EMBL-EBI website (http://www.ebi.ac.uk/Tools/msa/clustalo/). Alignments were manually checked for correct amino acid positioning at gaps. Anomalies were corrected and statistics recalculated as required. Maximum-likelihood phylogenetic trees were generated using software available at the website http://phylogeny.lirmm.fr/phylo_cgi/index.cgi [13]. The options used to generate phylogenetic trees were: initial sequence alignment—muscle; alignment curation—Gblock; phylogeny—PhyML; tree rendering—TreeDyn.

## 3. Results and discussion

The availability of high-quality genomic sequence for a large number of mammalian species in the ENSEMBL and Pre ENSEMBL databases has facilitated genomic analysis and the study of comparative genomics. This, in turn, has led to a new molecular-based understanding of evolution and phylogenies in particular. In this paper, the ENSEMBL databases have been used to elucidate the evolutionary history of UTP14 retrotransposition events that gave rise to intronless autosomal copies of this X-linked gene. These retrotransposed copies of UTP14 appear to have acquired an essential role in spermatogenesis with expression being limited to the male testis and no other tissue in either the male or female of a species[9–11]. Whereas the progenitor gene generally consists of 15 exons, retrotransposed copies contain a single exon that can be easily identified within mammalian genomes using simple search strategies such as BLAST. In addition, the presence of a contiguous coding frame allows for the identification of mutations that render the open reading frame functionally inoperative.

Table 1 documents the presence or absence of UTP14 retrogenes within the mammalian genomes currently available on the ENSEMBL genomic database [14]. The taxonomic groupings are based on molecular phylogenetics that can also be found on the ENSEMBL database [14]. Two taxonomic groups, Protheria (egg-laying mammals) and Metatheria (marsupials), do not contain any retrotransposed copies of UTP14. By contrast, all major groups of placental mammals (Eutheria) contain at least one extant member that possesses a retrotransposed copy of UTP14. In cases where the ENSEMBL database has identified the retrotransposed copy of UTP14, the associated ENSEMBL gene number is given in table 1. ‘Unidentified' in the table indicates copies of UTP14 retrogenes that can be identified by a BLAST search but are yet to be documented in the ENSEMBL database. ‘Remnant' refers to autosomal retrogene copies of UTP14 that contain mutations which disrupt the open reading frame or where only fragments could be identified. Cases where BLAST searches failed to identify any sequence with similarity to UTP14 are indicated by ‘none'. Where possible, retrotransposed copies of UTP14 that were identified are mapped back to the syntenic region of the human genome. Either DNA sequence flanking the retrogene, or genes in the immediate vicinity of the retrogene were used as markers for mapping back to the human genome. This is proof that these retrogenes are the result of independent retrotransposition events with distinct genomic insertion points. In several cases, there was insufficient data available to identify any synteny, and this is denoted by ‘N/I'. No species-specific chromosome assignments are presented in the table because, in the majority of cases, there is no information available that links genomic sequence to a species-specific chromosome.

**Table 1.** UTP14 retrogenes present in the genomes of species within the class Mammalia. The taxonomic groups in the first column are divided into Protheria (egg-laying mammals), Metatheria (marsupials) and Eutheria (placental mammals). Eutheria is further subdivided into the suborder Xenarthra, order Eulipotyphla, and clades Afrotheria, Scrotifera, Euarchonta and Glires. Individual species within these groups are listed by their common name. Presence or absence of UTP14 retrogenes within the genomes of the various species is listed in the third column. If no UTP14 retrogenes were identified within a genome, this fact is indicated as ‘none' in the table. If a UTP14 retrogene is present within a genome and has been listed on the ENSEMBL database, the ENSEMBL gene number is given. In cases where an intact UTP14 retrogene open reading frame was identified within a genome, but is yet to be documented by ENSEMBL, this is indicated by ‘unidentified'. In many cases non-functional copies of UTP14 retrogenes were also identified and these are listed as ‘remnant's. The fourth column lists the nearest adjacent gene and syntenic human chromosome in cases where this information could be identified. A situation where there is insufficient genomic data within the ENSEMBL database to identify a known adjacent gene or to determine any human synteny is indicated by ‘N/I’ (not identified).

taxonomic group	species	UTP14 retrogene	syntenic human chromosome
**Protheria**	platypus	none	
**Metatheria**	Tasmanian devil	none	
	wallaby	none	
	opossum	none	
**Eutheria**			
Xenarthra	sloth	ENSCHOG00000005754	N/I
		ENSCHOG00000009675	N/I
		ENSCHOG00000001479	N/I
		remnant	N/I
	armadillo	remnant	Ch. 15- STRAD9
Afrotheria	aardvark	ENSP00000428619	Ch. 11- ZDHHC24
	lesser hedgehog	remnant	N/I
	elephant	ENSLAFG00000027486	Ch. 11- ZDHHC24
	hyrax	remnant	Ch. 11- ZDHHC24
Eulipotyphla	hedgehog	remnant	Ch. 5- PCYOX1 L
	shrew	ENSSARG0000001254	N/I
		remnant	N/I
Scrotifera	pig	none	
	alpaca	none	
	sperm whale	none	
	dolphin	ENSP0000037794	Ch. 2- Linc RNA
	cow	ENSBTAG00000018708	Ch. 14- CATSPERB
		remnant	Ch. 6- MAP3K7
	sheep	ENSOARG00000012140	Ch. 14- CATSPERB
		remnant	Ch. 6- MAP3K7
	microbat	ENSMLUG00000005692	Ch. 9- RAD23B
		remnant	Ch. 15- IND80
	megabat	none	
	rhinoceros	none	
	horse	none	
	cat	none	
	dog	none	
	panda	none	
	ferret	none	
Euarchonta	tree shrew	remnant	Ch. 8- VPS13B
		remnant	Ch. 11- PGAP2
	mouse lemur	unidentified	Ch. 12- H2AFJ
	bush baby	none	
	tarsier	unidentified	N/I
	squirrel monkey	ENSP00000377944	Ch. 9- CTSL
		remnant	Ch.12- GXYLT1
	marmoset	ENSCJAG00000008114	Ch. 13- ALG11
	macaque	ENSMMUG00000016517	Ch. 13- ALG11
	olive baboon	ENSPANG00000004998	Ch. 13- ALG11
	vervet	ENSCSAG00000018620	Ch. 13- ALG11
	gibbon	ENSNLEG00000018242	Ch. 13- ALG11
	orangutan	unidentified	Ch. 13-ALG11
	gorilla	ENSGGOG00000034934	Ch. 13- ALG11
	chimpanzee	unidentified	Ch. 13- ALG11
	human	ENAG00000253797	Ch. 13- ALG11
Glires	pika	none	
	rabbit	unidentified	Ch. 14- STON2
	guinea pig	unidentified	Ch. 10- ZNF248
	naked mole rat	PREHGLG00000020293	Ch. 10- ZNF248
		remnant	Ch. 5- SEPT8
		remnant	Ch. 22- SLC25A1
		remnant	Ch. 6- EXOC2
	kangaroo rat	ENSDORG00000002224	N/I
	prairie vole	ENSP00000350012	Ch. 2- ACSL3
	mouse	ENSMUSG00000079470	Ch. 2- ACSL3
		remnant	Ch. 1- AMY1C
	rat	AABR07068127	Ch. 2- ACSL3
	Chinese hamster	unidentified	N/I
	squirrel	none	

### 3.1. Xenarthra

**Figure 1.**
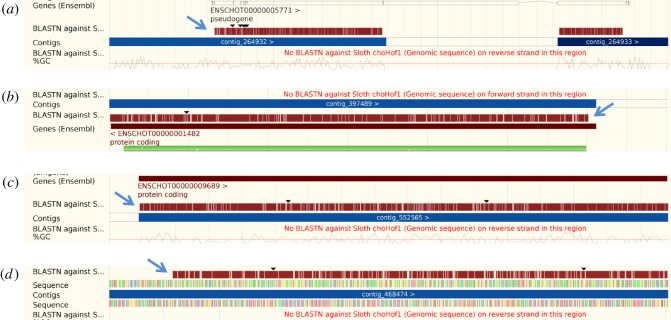
ENSEMBL BLAST alignments showing the position of UTP14 retrogene sequence within the sloth genome that was obtained using human UTP14A as the reference sequence. (*a*) Alignment of the reference sequence on scaffold 44 506 is indicated by the blue arrow. A large section of sequence is missing from the alignment without which the functionality of the total retrogene cannot be assessed. The fragmented gene has been assigned as a pseudogene by the ENSEMBL database. Completion of the sloth genome in this region will in future resolve the question of gene function. (*b*–*d*) Alignment of the human UTP14A reference sequence with the sloth genome revealed three additional retrotransposition events that are indicated with blue arrows. Alignments (*a*) and (*b*) on scaffolds 148 267 and 84 741, respectively, have been previously recognized as protein coding within the ENSEMBL database. However, both predicted peptides are missing sequence at both termini, so that it is not clear if these are functional copies or not. (*d*) The BLAST alignment identifying the novel retrotransposition of UTP14 on scaffold 111 529 that contains multiple mutations which render this copy non-functional. The presence of multiple copies of UTP14 retrogenes within the sloth genome is an indication of the unusually high frequency with which the UTP14 gene has retrotransposed within this lineage.

The genomes of two species within mammalian group Xenarthra are currently available. The sloth (*Choloepus hoffmanni*) genome contains one retrotransposed copy of UTP14 that constitutes a single open reading frame, covering the amino and carboxyl termini, but is missing some internal sequence data (figure 1). This is presumably an intact copy of UTP14 and is identified as a pseudogene on the ENSEMBL database (gene number ENSCHOG00000005754: scaffold 44 506). However, until the sequence gap is filled, intactness remains an open question. Additionally, there are three other copies of UTP14 that have disrupted open reading frames that make the genes translationally incomplete. Two of these copies contain a long open reading frame that when translated are missing the amino and carboxyl terminal amino acids. This is due to premature termination of the available scaffold sequence. These are designated as ENSEMBL genes ENSCHOG00000009675 (scaffold 148 267) and ENSCHOG00000001479 (scaffold 84 741). The fourth copy of UTP14 is located on sloth scaffold 111 529: 4317-5755 and consists of an open reading frame disrupted by multiple deletions and stop codons.

By contrast, the armadillo (*Dasypus novemcinctus*) genome contains a single disrupted copy of UTP14 within the third intron of the gene STARD9 covering 1600 bases. STARD9 is located on armadillo genome scaffold JH569607. The equivalent position in the human genome is on chromosome 15. This would indicate that there is a significant difference between the genomes of the sloth and armadillo in terms of UTP14 retrotransposition history. It is clear that UTP14 retrotransposition has occurred multiple times within this lineage and many of these copies have accumulated mutations within the open reading frame, thus rendering them inactive. Further work is needed to determine if these modified UTP14 retrogenes are still expressed in the testis.

### 3.2. Afrotheria

**Figure 2.**
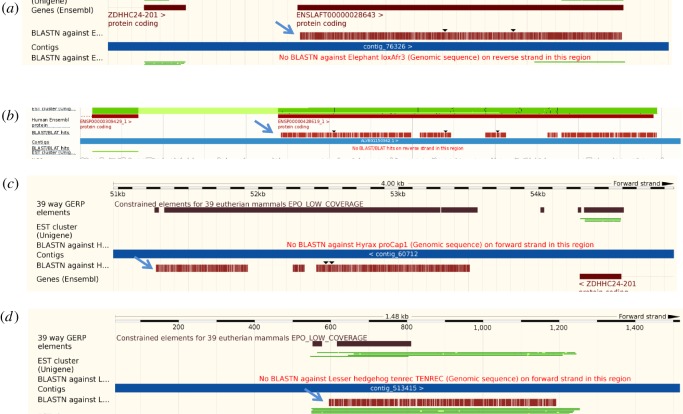
BLAST alignment of the human UTP14A reference sequence against the genomes of species belonging to the group Afrotheria that are available on the ENSEMBL database. The elephant (*a*), aardvark (*b*) and hyrax (*c*) genomes all contain a single retrotransposed copy of UTP14 that is located adjacent to the gene ZDHHC24. This indicates that in each of these species the UTP14 retrogene arose from a single ancestral event. In the case of the lesser hedgehog (*d*) a single retrotransposed copy of UTP14 was identified. There is insufficient sequence data available for this genome to determine if this copy is homologous to that present in the other members of Afrotheria.

Four genomes are available within the clade Afrotheria. The African elephant (*Loxodonta africana*) and aardvark (*Orycteropus afer afer*) both contain an intact retrotransposed copy of UTP14. By contrast, the hyrax (*Procavia capensis*) and lesser hedgehog (*Echinops telfairi*) contain remnants of a UTP14 retrogene (figure 2). In the case of the aardvark, elephant and hyrax, the UTP14 retrogene is located near the gene ZDHHC24 and is syntenic to a region on human chromosome 11. The intact aardvark retrogene is located on gene scaffold JH864772 and is listed as novel transcript number ENSP00000428619. In the elephant, the retrogene is located on scaffold 71 and designated as gene number ENSLAFG00000027486. A remnant UTP14 retrogene is present within the hyrax genome on scaffold 7640. The lesser hedgehog genome also contains a UTP14 retrogene fragment on scaffold 77 921. In both of these latter cases, the UTP14 retrogene remnants have yet to be identified by the ENSEMBL database. In the case of the lesser hedgehog, there is insufficient sequence available on scaffold 77 921 to identify a syntenic region of the human genome. The Afrotheria clade contains two major branches; the Afroinsectiphilia (containing the aardvark and lesser hedgehog) and Paenungulata (containing the elephant and hyrax). These two groups appear to have evolved in isolation on the African continent between 105 and 40 million years ago (Ma) [15] and may have diverged over 50 Ma. The presence of a single retrotransposed copy of UTP14 adjacent to the gene ZDHHC24 in members of both Afroinsectiphilia and Paenungulata would indicate that this retrogene became fixed in a common ancestor shared by these two lineages and has persisted in a diverse group of species for over 50 Myr. Over this time, there has been significant divergence between the elephant and aardvark UTP14 encoded peptide sequences (electronic supplementary material, figure S1). The absence of any retrogene sequence adjacent to ZDHHC24 in other mammalian groups suggests that the retrotransposition event that created this UTP14 retrogene is unique to the Afrotherian lineage and may be a characteristic feature of this lineage.

### 3.3. Eulipotyphla

Within the order Eulipotyphla, high-quality sequence data are available for the hedgehog (*Erinaceus europaeus*) and shrew (*Sorex araneus*). Both species carry retrogene copies of UTP14 within their genomes (table 1). Hedgehog carries a remnant retrogene on scaffold 7150 in front of the gene PCYOX1 L which is syntenic to a region on human chromosome 5. By contrast, the shrew genome contains an intact copy of a UTP14 retrogene (ENSSARG00000012854) on scaffold 12 854. A BLAST search also identified a UTP14 retrogene fragment on shrew scaffold 4297. Unfortunately, there is insufficient sequence data available within the two shrew genome scaffolds containing UTP14 retrogene sequence to identify any human synteny.

### 3.4. Scrotifera

Scrotifera is a large clade of mammals containing bats, carnivores, ungulates and cetaceans which modern molecular phylogenetics has shown to have arisen from a common ancestor. Most members of this clade do not carry any evidence of a UTP14 retrogene being present within their genome (table 1). Currently, there are four members of Scrotifera within whose genome UTP14 retrogenes can be identified. The dolphin (*Tursiops truncatus*) has a single copy that appears to be intact on scaffold JH474539, and has been assigned gene number ENSP0000037794. This retrogene maps back to a syntenic region of human chromosome 2 adjacent to Linc RNA AC096649. The microbat (*Myotis lucifungus*) from North America has a single intact UTP14 retrogene copy located on scaffold GL429810, which has the gene designation ENSMLUG00000005692. It is located between the genes RAD23B and ZNF462 that map to human chromosome 9. In addition, there is a second disrupted UTP14 retrogene copy on scaffold GL429876 between the genes IND80 and EXD1 which maps to a syntenic region on human chromosome 15. By contrast, the megabat (*Pteropus vampyrus*) from East Asia does not contain any retrotransposed copies of UTP14. These data would suggest that, in some species of bats, independent retrotransposition events have resulted in the accumulation of multiple UTP14 retrogene copies that may be potentially useful as phylogenetic markers.

**Figure 3.**
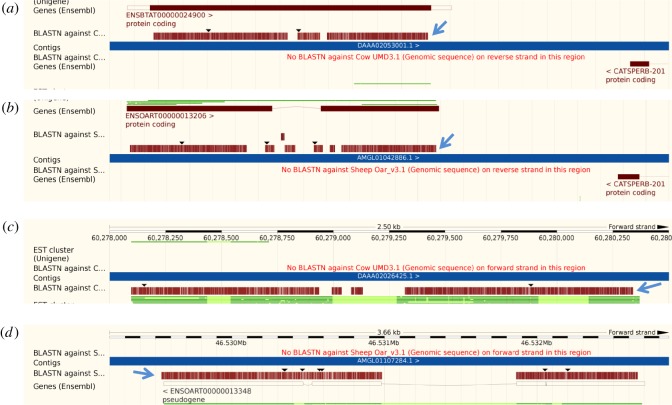
UTP14 retrogenes located within the cow and sheep genomes. Both the cow and sheep contain two homologous copies of UTP14 retrogenes that can be identified by homology with human UTP14A reference sequence using the ENSEMBL BLAST function. BLAST alignments are indicated by blue arrows. Cow possesses an intact protein coding copy of UTP14 adjacent to the gene CATSPERB that is shown in (*a*). The sheep equivalent that is disrupted by an intron is shown in (*b*). In addition, both cow (*c*) and sheep (*d*) contain a second retrotransposed copy of UTP14 that has not previously been identified in the cow but is curated as a pseudogene in the sheep. In both cases, the second copy contains numerous mutations that render the genes translationally inactive.

Of special interest is the evolutionary history of UTP14 retrogenes in the cow (*Bos taurus*) and sheep (*Ovis aries*). Both these species possess two analogous UTP14 retrogene copies within their genomes. The cow has a single intact copy (ENSBTAG00000018708) behind the gene CATSPERB that maps to human chromosome 14 (figure 3*a*). There is also an inactive remnant in front of MAP3K7 which is not recognized within the ENSEMBL database and maps to human chromosome 6 (figure 3*c*). In the sheep, the UTP14 retrogene behind CATSPERB (ENSOARG00000012140) is disrupted by an intron on the ENSEMBL database (figure 3*b*). However, a BLAST search indicated that this intron contains a high level of homology to the UTP14 sequence. For this reason, it is suspected that this copy of UTP14 is either inactive or its function has been altered by the acquisition of an intron. The second sheep UTP14 retrogene in front of MAP3K7 (ENSOARG00000012276) is listed as a pseudogene in the ENSEMBL database (figure 3*d*). It is disrupted by two introns and multiple frameshifting deletions that clearly render this gene non-functional. It would appear that a common ancestor shared by both cow and sheep over 18 Ma [16] possessed two UTP14 retrogenes. However, in the cow one copy has maintained function, whereas both copies in the sheep have been extensively modified making their functional intactness questionable. There is a possibility that the copy adjacent to CATSPERB in sheep is a functional but internally truncated copy of UTP14. Further work is needed to establish if this is indeed the case.

### 3.5. Euarchonta

The next major group listed in table 1 is the grandorder Euarchonta that contains tree shrews, flying lemurs and primates. The tree shrew (*Tupaia belangeri*) belongs to the order Scandentia and its genomic sequence contains two UTP14 retrogenes. The first is on scaffold 2303 within an intron of the gene VPS13B that maps to human chromosome 8. This copy lacks the 5′ half of the parental gene and contains multiple deletions that result in frameshifts rendering the gene inactive. This copy is not recognized within the ENSEMBL database. A second copy is located on scaffold 3177 in front of the gene PGAP2 and is listed as a pseudogene (ENSTBEG00000000721). It maps to human chromosome 11 and is disrupted by an intron. No members of the order Dermoptera, which contains flying lemurs, have been sequenced to date. By contrast, the Primate order is well represented within the ENSEMBL database. The bush baby (*Otolemur garnettii*) is the only sequenced member of this group that does not possess a UTP14 retrogene. By contrast, its close relative, the mouse lemur (*Microbus murinus*), has a complete and potentially fully functional retrotransposed copy of UTP14 on scaffold 4464. This retrogene is in front of the gene H2AFJ that maps to human chromosome 12. This retrogene is yet to be identified within ENSEMBL. The tarsier (*Tarsius syrichta*) lies outside of the group containing monkeys and apes. Available tarsier genomic sequence consists of a large number of short isolated scaffolds, two of which contain parts of a UTP14 retrogene. The first 400-plus bases of a UTP14 retrogene are present on the 5′ end of scaffold 81 620 and the remainder is located on the 3′ end of scaffold 91 403. A BLAST search indicated that the tarsier retrogene sequence is largely intact, suggesting that the two scaffolds may eventually map adjacent to each other and potentially contain two fragments of a functional retrogene. Completion of the tarsier genome will be needed before this question can be resolved. Because of the fragmented nature of the scaffolds containing the UTP14 retrogene sequence, mapping back to the human genome was not possible.

Monkeys and apes form the other groups within the primate lineage for which high-quality genomic sequence is available on ENSEMBL. All members of these two groups contain UTP14 retrogenes. Most contain a single copy that maps to human chromosome 13, which presumably arose in a common ancestor. Only the New World squirrel monkey (*Samiri boliviensis*) is an exception with two unique UTP14 retrogenes. Because monkeys and apes share retrogenes of common ancestry that contain lineage-specific modifications, it is possible to do a simple cladistics analysis that allows inference of gene-specific phylogenies within this primate group.

**Figure 4.**
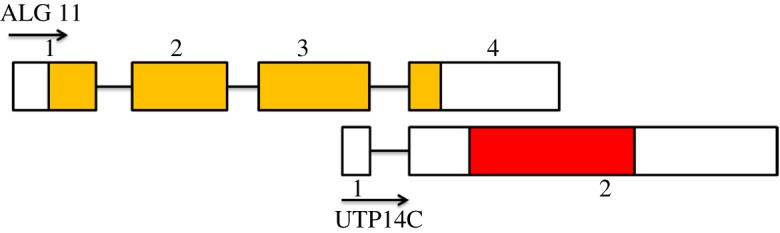
The primate retrogene UTP14C is located within the gene ALG11. UTP14C is a retrotransposed copy of the X-linked gene UTP14A which is inserted downstream from the coding sequence within the terminal exon of ALG11. The coding sequence of ALG11 (yellow) spans four exons. The coding sequence of UTP14C (red) overlaps with the 3′ UTR of its host gene. The transcriptional start site of UTP14C is located within the coding sequence making up exon three of ALG11. Exons three and four of ALG11 and one and two of UTP14C share the same splice site used to produce the mature mRNAs. ALG11 is ubiquitously expressed in all tissues, whereas UTP14C is expressed only in the spermatogenic tubules of the male testis.

In the case of monkeys and apes, the parental X-linked copy of UTP14 has been designated as UTP14A, whereas the retrotransposed copy mapping to human chromosome 13 is known as UTP14C [11]. Primate UTP14C consists of two exons. The 5′ non-coding exon overlaps with the coding region of the host gene ALG11 exon 3 [11] (figure 4). The terminal 3′ exon of UTP14C contains the complete sequence of the terminal exon (exon 4) of ALG11 as well as an uninterrupted open reading frame encoding a peptide with high homology to that encoded by UTP14A. The splice junction which unites ALG11 exons 3 and 4 is shared by the two exons of UTP14C. With this arrangement, ALG11 produces a ubiquitously expressed transcript that is minus the retrotranspositioned coding region of UTP14C. In contrast, UTP14C is expressed only in the testis as a single open reading frame [11]. Testis-specific expression of UTP14C must confer some selective advantage as it has survived in primate lineages for millions of years. This unusual arrangement is present within the genomes of some New World monkeys (Platyrrhini) and all Old World primates (Catarrhini). However, it is absent in tarsiers, lemurs and bush babies, suggesting that UTP14C arose specifically in the lineage that gave rise to Old World and New World primates. The location of UTP14C within ALG11, and the fact that it contains a single large open reading frame, facilitates its identification and compilation within the various primate lineages; even when the ENSEMBL database lacks clear annotation.

**Figure 5.**
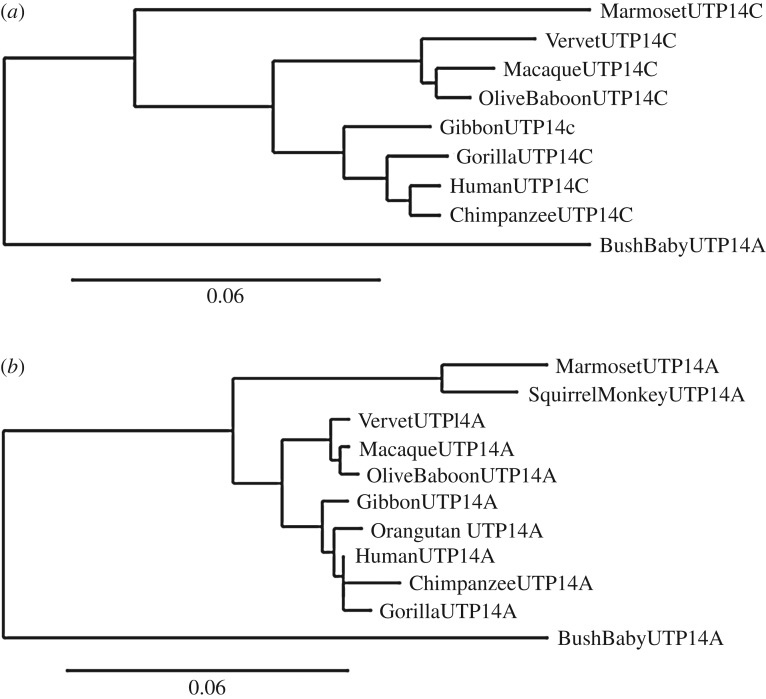
Primate evolutionary trees based on the peptide sequence of UTP14A and its daughter retrogene UTP14C. (*a*) The maximum-likelihood evolutionary tree for UTP14C based on sequence data available on the ENSEMBL database. Bush baby UTP14A was used to anchor the tree because it is basal to any retrotransposition event that led to the creation of UTP14C. (*b*) The maximum-likelihood tree based on the coding sequence for UTP14A currently available on the ENSEMBL Genome Database. Bush baby sequence was used to anchor the tree. Divergence of the New World monkeys (squirrel monkey and marmoset) preceded that of the Old World monkeys (vervet, macaque and olive baboon), which in turn preceded the divergence of lesser apes (gibbon) and great apes (orangutan, gorilla, chimpanzee and human). This pattern of UTP14A evolution follows the generally accepted pattern of primate evolution. The observed branching for UTP14C is similar to that observed for UTP14A, leading to the conclusion that the pattern of UTP14C evolution is similar to that generally observed in primates even though it is subject to unique selective pressures because of its function in male spermatogenesis. In (*a*,*b*), the branch length is proportional to the number of substitutions per site. Orangutan was omitted from the UTP14C analysis because the version of UTP14C it carries contains a large deletion as well as many nucleotide substitutions that render it unsuitable for phylogenic analysis.

To perform a robust phylogenetic analysis, it was important to establish that UTP14C has accumulated amino acid variants in a manner consistent with existing primate phylogenies. The family tree generated for UTP14C (figure 5*a*) was almost identical to that for the progenitor gene, UTP14A, (figure 5*b*) and the accepted primate phylogeny [17]. This establishes that even though UTP14C is expressed specifically in the testis, it is subject to genetic drift in a manner that does not differ significantly from that of other primate genes and its parental gene in particular. It should be noted that the orangutan was left out of the analysis presented in figure 5*b* because the first 1200 bases of UTP14C coding sequence have been deleted in the orangutan (*Pongo abelii*) genome (electronic supplementary material, figure S2) and the remaining sequence has acquired multiple mutations that affect the remaining open reading frame. This deletion has not affected the host gene ALG11. It remains to be determined if the truncated form of UTP14C is still transcribed in the orangutan testis.

**Figure 6.**
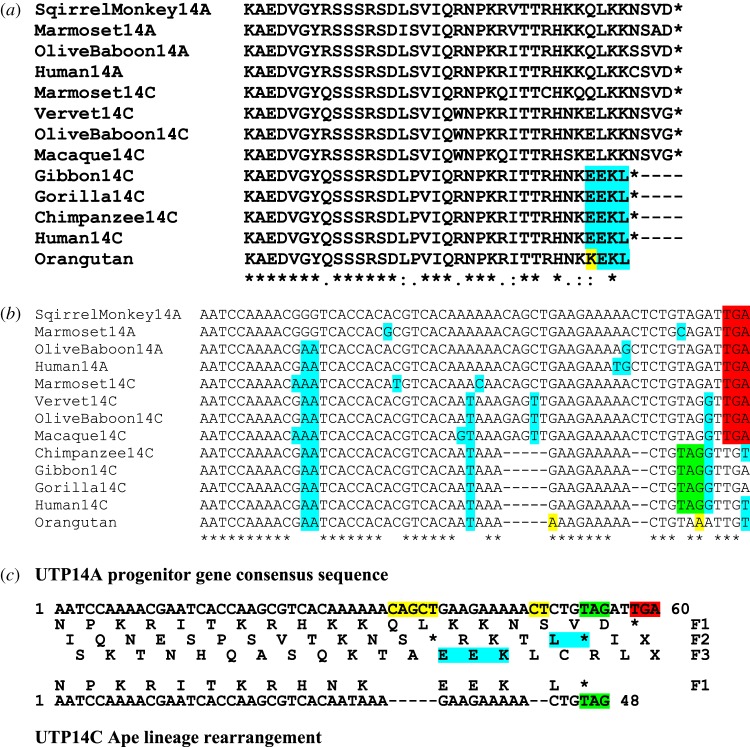
Lineage-specific variation in the terminal amino acid sequences of UTP14A and C. (*a*) The alignment of the carboxyl terminus of UTP14A and C. The terminal amino acid sequence is highly conserved in all primate groups. UTP14C proteins from the Old World monkeys (vervet, olive baboon and macaque) and the New World marmoset monkey share amino acid homology with that present in the progenitor UTP14A protein terminus. The ape family, represented here by the gibbon (lesser ape) together with the orangutan, gorilla, chimpanzee and human (great apes), possesses an alternative version of UTP14C where the carboxyl terminus has been modified. The altered carboxyl terminus shared by the ape lineage is highlighted in blue. An orangutan-specific amino acid change is highlighted in yellow. An analysis of the DNA sequence encoding the terminal amino acids of UTP14A and C (*b*) shows that the modification of the carboxyl terminus of UTP14C in the ape lineage is due to two tandem deletions of five and two bases. Nucleotide changes are highlighted in blue, ancestral stop codons are red, ape-specific stop codons are indicated in green and orangutan-specific nucleotide changes are highlighted in yellow. The effect of the tandem nucleotide deletions within the ape lineage is illustrated in (*c*). The upper nucleotide sequence is that of the ancestral UTP14A that gave rise to UTP14C by retrotransposition. The lower sequence is that of ape UTP14C. The bases lost from the original sequence are highlighted in yellow. The translation products of the three forward open reading frames of UTP14A are also shown. The first five base-pair deletion results in a reading frameshift (to F3) and the incorporation of three alternative amino acids (blue). A downstream two-base deletion results in another frameshift (to F2) so that a single amino acid is replaced (blue) and a previously silent out-of-frame stop codon (green) is brought into frame, thus terminating translation of the modified ape UTP14C. The original stop codon used by UTP14A is highlighted in red.

Of particular interest is a lineage-specific variation in the carboxyl terminus of UTP14C that is clearly identified when the terminal 39 amino acids from the various UTP14A and C variants present in New World and Old World primates are aligned. The amino acid terminus of UTP14C in the Old World monkeys, vervet (*Chlorocebus sabaeus*), macaque (*Macaca mulatta*) and olive baboon (*Papio anubis*) as well as the New World marmoset monkey (*Callithrix jacchus*) is homologous to that of the ancestral primate UTP14A from which UTP14C originated (figure 6*a*). By contrast, the Old World apes, gibbon (*Nomascus leucogenys*), orangutan (*Pongo abelii*), gorilla (*Gorilla gorilla gorilla*), chimpanzee (*Pan troglodytes*) and human (*Homo sapiens*) share a version of UTP14C whose terminal amino acid sequence differs significantly from that of UTP14A and monkey UTP14C (figure 6*a*). This amino acid difference comprises the loss of four terminal amino acids and a cluster of conserved changes in what are now the six terminal amino acids of the modified UTP14C. The alteration in UTP14C is shared by all Old World apes and appears to have been highly conserved since the ape lineage diverged from monkeys over 25 million years ago and may thus be a potential molecular marker for this lineage [18].

An analysis of the coding sequence that translates into the terminal amino acids of ape UTP14C is presented in figure 6*b*. Alignment of the relevant sequences reveals that two tandem deletions are responsible for the amino acid change observed in Old World apes. The first is a five-nucleotide deletion that results in the loss of two amino acids and a frameshift so that open reading frame three now contributes the next three amino acids. A second deletion of two nucleotides results in another frameshift that brings frame two of the original unaltered sequence into translational alignment so that a single amino acid is substituted before a previously inactive stop codon in frame two terminates translation (figure 6*c*). This rather unusual arrangement of two tandem deletions can be regarded as a very rare event that has been fixed in the ape lineage and therefore can be considered to have an important selective advantage via its influence on spermatogenesis in this lineage. It may also represent an instance where modification of a highly conserved peptide confers some form of functional specificity to the new variant that was suboptimal in the UTP14C monkey homologues that carry the parental pattern of amino acids.

**Figure 7.**
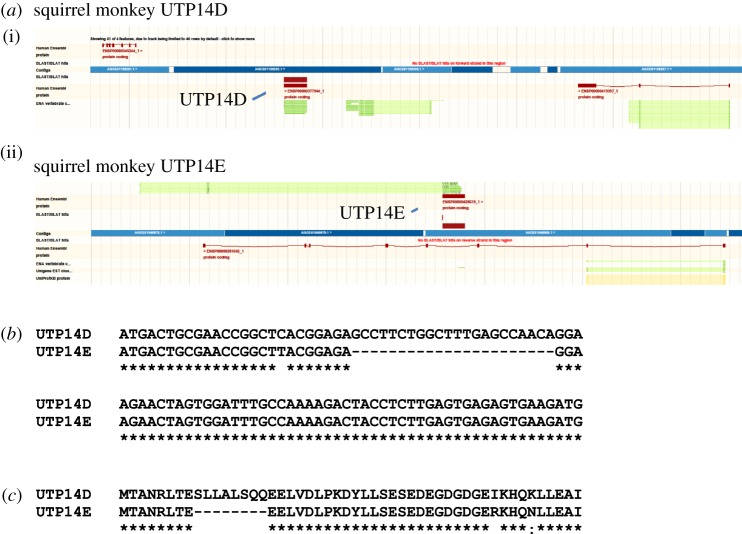
Squirrel monkey genome contains two novel retrogenes, UTP14D and E. (*a*) The Pre ENSEMBL genomic scaffolds that contain the squirrel monkey retrogenes. UTP14D (i) is located on squirrel monkey scaffold JH378246.1 between the upstream gene CTSL (ENSP00000345344_1) and the downstream gene ZNF782 (ENSP00000419397_1). UTP14D consists of a single intronless open reading frame (ENSP00000377944_1) which codes for a peptide homologous to UTP14A. UTP14E (ii) is located on squirrel monkey scaffold JH378123.1 within the third intron of GXYLT1 (ENSP00000381666_1). UTP14E consists of a single intronless open reading frame (ENSP00000428619_1). (*b*) The alignment of the 5′ coding sequence of UTP14D and E that contains the 22 base-pair deletion within UTP14E which causes a frameshift that disrupts the open reading frame. (*c*) The translation of the DNA sequence presented in (*b*) that documents the amino acids lost from UTP14E due to the deletion. The amino acid sequence shown beyond the deletion in UTP14E is from open reading frame 3 which contains the remaining complete intact coding sequence of UTP14E.

Although the squirrel monkey genome contains a fully functional copy of ALG11, which is almost identical to that present in marmoset, there is a marked absence of any sequence that can be attributed to UTP14C in the region immediately upstream of ALG11 within the squirrel monkey genome (electronic supplementary material, figure S3). There are three possible explanations for this observation. In the squirrel monkey, there is a possibility that the region upstream of the coding sequence in the terminal exon of ALG11 has never contained a copy of UTP14C. Alternatively, UTP14C has either completely degraded to the point that it is no longer recognizable or the gene has been deleted by precise removal [19]. However, the squirrel monkey genome does contain two retrotransposed copies of UTP14A that appear to be unique. These two copies have been designated as primate UTP14D and E. The first copy, UTP14D, appears to be fully functional and consists of a single contiguous open reading frame located between the two genes CTSL and ZNF782 that are syntenic to a region on human chromosome 9 (figure 7*a*). UTP14E is located within the third intron of the gene GXYLT1 that maps to human chromosome 12. The open reading frame of UTP14E contains a 22 base-pair deletion that renders the gene non-functional due to a frameshift (figure 7*b,c*; electronic supplementary material, figure S4). Interestingly, the open reading frame 3 immediately downstream from the deletion is predicted to code for the remainder of an intact UTP14 peptide that is disrupted by a single internal stop codon (electronic supplementary material, figure S5). This would suggest that the inactivation of UTP14E is a recent and ongoing process. Alignment of squirrel monkey UTP14A, D and E coding sequence indicates that UTP14D is the more ancient of the two retrotransposition events as it carries 33 unique nucleotide substitutions not shared with either UTP14A or E (electronic supplementary material, figure S4). By contrast, UTP14E contains 13 unique nucleotide substitutions in addition to the large deletion which renders the gene translationally redundant. This is consistent with the hypothesis that it represents a more recent retrotransposition event that is currently under negative selective pressure. The ubiquitously expressed UTP14A contains eight unique nucleotide substitutions, indicating that it is subject to more stringent selective pressures than either of its daughter retrogenes.

### 3.6. Glires

The clade Glires contains two orders, Lagomorpha and Rodentia. The ENSEMBL database contains genomic sequence for two members of Lagomorpha, the pika (*Ochotona princeps*) and rabbit (*Oryctolagus cuniculus*). The pika genome lacks any trace of a UTP14 retrogene (table 1). However, the rabbit genome contains an unidentified retrogene on chromosome 20 between the genes STON2 and GTF2A that maps to a syntenic region on human chromosome 14.

**Figure 8.**
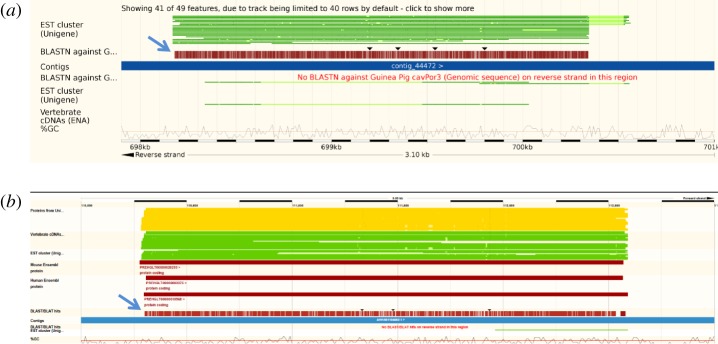
UTP14 retrogenes identified within the genomes of the guinea pig and naked mole rat. BLAST alignment of the human UTP14A reference sequence against the genomes of the guinea pig (*a*) and naked mole rat (*b*) is indicated by blue arrows. In both cases, the retrogene is located in front of the gene ZNF248 which indicates that they are homologous.

Eight members of the order Rodentia are represented within the ENSEMBL database and nearly all contain copies of UTP14 retrogenes. The squirrel (*Ictidomys tridecemlineatus*), which belongs to the suborder Sciuromorpha, does not possess a UTP14 retrogene. Guinea pig (*Cavia porcellus*) and naked mole rat (*Heterocephalus glaber*) belong to the suborder Hystricomorpha and share homologous copies of a UTP14 retrogene (figure 8). In the guinea pig, a previously unidentified intact copy of UTP14 is located on scaffold 62 in front of the gene ZNF248 that is syntenic with human chromosome 10. In the naked mole rat, this gene homologue is located on scaffold JH602235 and has been assigned gene number PREHGLG00000020293. In addition, the naked mole rat also possesses three other retrotransposed copies of UTP14 (electronic supplementary material, figure S6). The first is a fragment located on scaffold JH602046 between the genes SEPT8 and KIF3A that map to human chromosome 5. Multiple fragments of a UTP14 retrogene are present within scaffold JH602258 near the gene SLC25A1 that maps to human chromosome 22. There is also a large UTP14 retrogene fragment of approximately 2 kb on scaffold JH602069 upstream of the gene EXOC2 that is syntenic with human chromosome 6. As both the guinea pig and naked mole rat possess UTP14 retrogenes located near the gene ZNF248, these retrogenes may be a taxonomic feature of the suborder Hystricomorpha. This is of particular interest as guinea pigs have evolved in the South American Andes, whereas the naked mole rat is found only in East Africa. This indicates a strong probability that this retrogene was fixed in a common ancestor that predated the separation of the South American and African continents.

**Figure 9.**
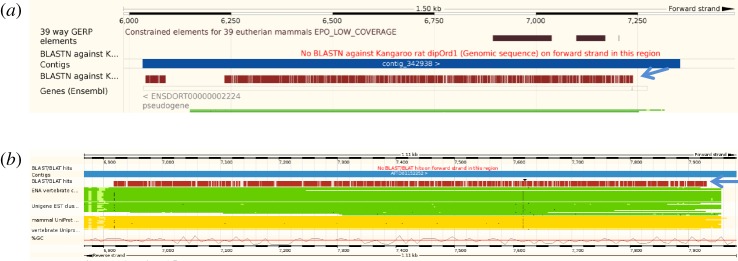
UTP14 retrogenes located within the genomes of kangaroo rat and Chinese hamster. (*a*) The alignment of the human UTP14A reference sequence with the kangaroo rat pseudogene on scaffold 37 991 (indicated by the blue arrow). This UTP14 retrogene is extensively fragmented with no evidence of a functional open reading frame. (*b*) Illustrates alignment of the human UTP14A reference sequence with a region on Chinese hamster scaffold JH005599 that contains an intact copy of a UTP14 retrogene. The BLAST alignment is indicated by the blue arrow.

**Figure 10.**
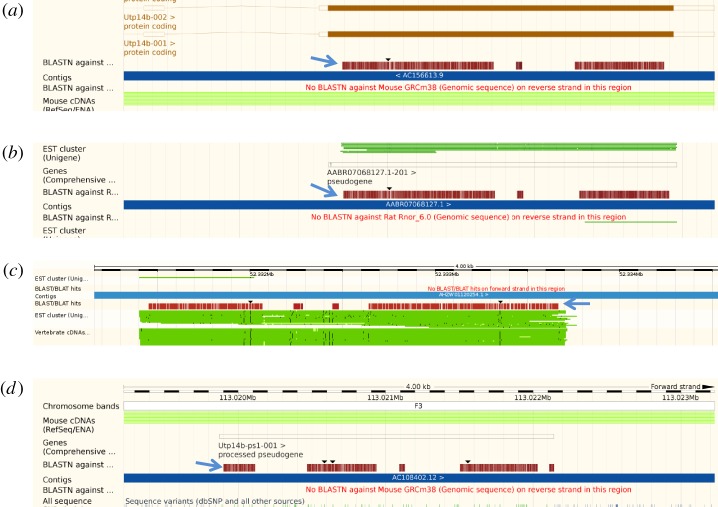
The known retrogene, UTP14b, is present within the genomes of the mouse, rat and prairie vole. Alignment of the human UTP14A reference sequence within the genomes of (*a*) mouse, (*b*) rat and (*c*) prairie vole is indicated by the blue arrows. All three species contain an intact reading frame which suggests that these retrogenes can code for functional proteins in all three species. The UTP14b retrogene copy present in the prairie vole is yet to be identified by ENSEMBL. (*d*) The alignment of the human UTP14A reference sequence with the mouse UTP14 pseudogene present within the mouse genome.

The kangaroo rat (*Dipodomys ordii*), which belongs to the suborder Castorimorpha, has an intronless copy of UTP14 on scaffold 37 991, which is identified as pseudogene gene number ENSDORG0000002224 located near the gene RPL21 and maps to human chromosome 13 (figure 9). Unfortunately, the genomic sequence spanning this pseudogene is fragmented due to missing sequence data, so it is not clear whether this gene really is a pseudogene, or a real gene which has been misassigned. The remaining four rodents belong to the suborder Myomorpha. Chinese hamster (*Cricetulus griseus*) contains what appears to be a complete open reading frame of a UTP14 retrogene on scaffold JH005599 which has yet to be annotated by ENSEMBL (figure 9). The scaffold containing this retrogene is small and lacks sufficient sequence for alignment with the human genome or determining its relationship with the retrogenes carried by other members of this group. The prairie vole (*Microtus ochrogaster*), mouse (*Mus musculus*) and rat (*Rattus norvegicus*), all possess homologous retrogenes that are located within the first intron of the gene ACSL3 [9,10] (figure 10) that has been officially designated as UTP14b in mouse and maps to a syntenic region on human chromosome 2. In the case of the rat, ENSEMBL has assigned this retrogene as the pseudogene AABR07068127 even though it consists of a large open reading frame. The prairie vole retrogene is located on chromosome LG4 and is yet to be recognized by ENSEMBL. An alignment of the UTP14 retrogene from all three species indicated that this gene has significantly diverged since these species shared a common ancestor over 22 million years ago [20] (electronic supplementary material, figure S6). In addition to UTP14b, the mouse genome also contains another copy of UTP14 that is a pseudogene which has been designated in ENSEMBL as UTP14b-ps1 (figure 10*d*). It is located near the gene AMY2a5 on mouse chromosome 3, which is syntenic with human chromosome 1.

## 4. Conclusion

It is thus clear that, throughout the evolution of eutherian mammals, UTP14 has been independently duplicated multiple times by the process of retrotransposition. In many cases, these UTP14 retrogenes show a history of lineage-specific conservation that spans millions of years of evolutionary history. In other cases, UTP14 retrogenes appear to have been rendered translationally inactive by the accumulation of mutations, as well as insertions and deletions, so all that remains are pseudogenes or fragments of retrogene-specific sequence. UTP14 retrogenes have previously been shown to be essential for spermatogenesis in humans [11] and mice [9,10], and hence, by analogy, it is presumed that these retrogenes may have an essential role for spermatogenesis in male mammals that carry functional copies of these retrogenes. In humans and mice, expression of UTP14 retrogenes is limited to the male testis and is absent in somatic tissues and the female reproductive tract. This would indicate that UTP14 retrogenes are under zero or negative selective pressure in females but under strong positive selective pressure in males. This functional specificity that limits the expression of UTP14 retrogenes to the male testis, and gametogenesis in particular implies the acquisition of specific promoters and regulatory elements after the initial retrotransposition event. Successful fixation of these genes, and their functional maintenance, are evidence for a complex series of evolutionary events that are both rare and lineage-specific. These retrogenes can thus give a rare insight into the evolutionary process that creates new genes which directly modulate spermatogenic efficiency and male reproductive success in many eutherian lineages.

Because retrotransposition and fixation of UTP14 retrogenes is a rare event that leads to structural variation within mammalian genomes [21], the possibility exists that their presence within mammalian genomes may be useful in resolving phylogenetic relationships. The utility of using rare genomic changes for phylogenetic analysis [22] is well established and retrotransposons have previously been used to establish eutherian phylogenies [23–25]. Currently, there is insufficient high-quality complete genome sequence data available to do statistically meaningful analyses for many taxonomic groups. However, this situation will improve as the genomes of more eutherian species are sequenced. Primates are the only group for which there are sufficient data to build robust phylogenies based on the UTP14 retrogene sequence. Presented herein is evidence that a set of tandem deletions within the 3′ coding sequence of UTP14C in the ape lineage distinguishes this lineage from other primates. In addition, there is the surprising observation that some New World monkeys appear to have diverged from other primates with respect to the UTP14 retrogenes present within their genomes. The marmoset genome contains a UTP14 retrogene that can be identified in all other extant Old World primate genomes currently sequenced. However, there is no sign of this retrogene within the squirrel monkey genome, where two unique retrotransposed copies have been identified. This difference may be potentially useful for future phylogenic analysis of New World monkey lineages, and effort should be expended to explore the diversity of UTP14 retrogenes within the New World monkeys. The lineage-specific loss of UTP14 retrogenes reported herein is the first documentation of this phenomenon. Previous work by Carelli *et al.* [26] involving genome-wide sampling of retrogenes across divergent phyla ‘could not detect any lineage-specific loss of retrogenes from testis-specific retrogene families' (quoted from page 306; Carelli *et al.* [26]). Owing to the importance of UTP14 retrogenes for successful male gametogenesis and reproduction in eutherian males, it would be useful to explore the functional role of UTP14 retrogene variants in spermatogenesis within different taxonomic groups. Furthermore, this may be a classic example of convergent recruitment where homologous gene duplication in different lineages is linked to a particular biological function. Future studies will undoubtedly lead to new insights into male gametogenesis and its role in reproductive success. Additionally, they will help to elucidate evolutionary paths that are driven by successful male reproduction.

## Supplementary Material

Fig S1. Divergence of UTP14 retrogene peptides encoded within the genomes of African elephant and aardvark.

## Supplementary Material

Fig S2. Half of orangutan UTP14C has been deleted without impacting the function of its host gene ALG11.

## Supplementary Material

Fig S3. Alignment of the terminal exon and flanking 3' sequence of ALG11 from the bush baby, human, marmoset and squirrel monkey identifies potential points of retrogene insertion and deletion.

## Supplementary Material

Fig S4. Alignment of squirrel monkey UTP14A and the retrogenes UTP14D and E reveals limited sequence divergence.

## Supplementary Material

Fig S5. Translation of squirrel monkey UTP14E indicates that this gene may have only relatively recently been inactivated.

## Supplementary Material

Fig S6. In addition to a functional copy of a UTP14 retrogene the naked mole rat genome caries three nonfunctional copies.

## Data accessibility

Sequence data used for database searches and generation of phylogenies have been deposited in the Dryad database (http://dx.doi.org/10.5061/dryad.250dt) [27].

## References

[1] Ramm SA, Scharer L, Ehmcke J, Wistuba J 2014 Sperm competition and the evolution of spermatogenesis. Mol. Hum. Reprod. 20, 1169–1179. (doi:10.1093/molehr/gau070) 25323971 10.1093/molehr/gau070

[2] Kaessmann H 2010 Origins, evolution, and phenotypic impact of new genes. Genome Res. 20, 1313–1326. (doi:10.1101/gr.101386.109) 20651121 10.1101/gr.101386.109 PMC2945180

[3] Long M, VanKuren NW, Chen S, Vibranovski MD 2013 New gene evolution: little did we know. Annu. Rev. Genet. 47, 307–333. (doi:10.1146/annurev-genet-111212-133301) 24050177 10.1146/annurev-genet-111212-133301 PMC4281893

[4] Chen S, Krinsky BH, Long M 2013 New genes as drivers of phenotypic evolution. Nat. Rev. Genet. 14, 645–660. (doi:10.1038/nrg3521) 23949544 10.1038/nrg3521 PMC4236023

[5] Vinckenbosch N, Dupanloup I, Kaessmann H 2006 Evolutionary fate of retroposed gene copies in the human genome. Proc. Natl Acad. Sci. USA 103, 3220–3225. (doi:10.1073/pnas.0511307103) 16492757 10.1073/pnas.0511307103 PMC1413932

[6] Daish TJ, Casey AE, Grutzner F 2015 Lack of sex chromosome specific meiotic silencing in platypus reveals origin of MSCI in therian mammals. BMC Biol. 13, 106 (doi:10.1186/s12915-015-0215-4) 26652719 10.1186/s12915-015-0215-4 PMC4676107

[7] Wang PJ 2004 X chromosomes, retrogenes and their role in male reproduction. Trends Endocrinol. Metab. 15, 79–83. (doi:10.1016/j.tem.2004.01.007) 15036254 10.1016/j.tem.2004.01.007

[8] Dragon F et al. 2002 A large nucleolar U3 ribonucleoprotein required for 18S ribosomal RNA biogenesis. Nature 417, 967–970. (doi:10.1038/nature00769) 12068309 10.1038/nature00769 PMC11487672

[9] Rohozinski J, Bishop CE 2004 The mouse juvenile spermatogonial depletion (jsd) phenotype is due to a mutation in the X-derived retrogene, mUtp14b. Proc. Natl Acad. Sci. USA 101, 11 695–11 700. (doi:10.1073/pnas.0401130101) 10.1073/pnas.0401130101 PMC511039 15289605

[10] Bradley J, Baltus A, Skaletsky H, Royce-Tolland M, Dewar K, Page DC 2004 An X-to-autosome retrogene is required for spermatogenesis in mice. Nat. Genet. 36, 872–876. (doi:10.1038/ng1390) 15258580 10.1038/ng1390

[11] Rohozinski J, Lamb DJ, Bishop CE 2006 UTP14c is a recently acquired retrogene associated with spermatogenesis and fertility in man. Biol. Reprod. 74, 644–651. (doi:10.1095/biolreprod.105.046698) 16354793 10.1095/biolreprod.105.046698

[12] Flicek P et al. 2014 Ensembl 2014. Nucleic Acids Res. 42, D749–D755. (doi:10.1093/nar/gkt1196) 24316576 10.1093/nar/gkt1196 PMC3964975

[13] Dereeper A et al. 2008 Phylogeny.fr: robust phylogenetic analysis for the non-specialist. Nucleic Acids Res. 36, W465–W469. (doi:10.1093/nar/gkn180) 18424797 10.1093/nar/gkn180 PMC2447785

[14] Yates A et al. 2016 Ensembl 2016. Nucleic Acids Res. 44, D710–D716. (doi:10.1093/nar/gkv1157) 26687719 10.1093/nar/gkv1157 PMC4702834

[15] Hedges SB 2001 Afrotheria: plate tectonics meets genomics. Proc. Natl Acad. Sci. USA 98, 1–2. (doi:10.1073/pnas.98.1.1) 11136239 10.1073/pnas.98.1.1 PMC33345

[16] Kato A, Rooney AP, Furutani Y, Hirose S 2010 Evolution of trappin genes in mammals. BMC Evol. Biol. 10, 31 (doi:10.1186/1471-2148-10-31) 20113469 10.1186/1471-2148-10-31 PMC2831891

[17] Perelman P et al. 2011 A molecular phylogeny of living primates. PLoS Genet. 7, e1001342 (doi:10.1371/journal.pgen.1001342) 21436896 10.1371/journal.pgen.1001342 PMC3060065

[18] Stevens NJ et al. 2013 Palaeontological evidence for an Oligocene divergence between Old World monkeys and apes. Nature 497, 611–614. (doi:10.1038/nature12161) 23676680 10.1038/nature12161

[19] van de Lagemaat LN, Gagnier L, Medstrand P, Mager DL 2005 Genomic deletions and precise removal of transposable elements mediated by short identical DNA segments in primates. Genome Res. 15, 1243–1249. (doi:10.1101/gr.3910705) 16140992 10.1101/gr.3910705 PMC1199538

[20] Steppan S, Adkins R, Anderson J 2004 Phylogeny and divergence-date estimates of rapid radiations in muroid rodents based on multiple nuclear genes. Syst. Biol. 53, 533–553. (doi:10.1080/10635150490468701) 15371245 10.1080/10635150490468701

[21] Ewing AD, Ballinger TJ, Earl D, Harris CC, Ding L, Wilson RK, Haussler D 2013 Retrotransposition of gene transcripts leads to structural variation in mammalian genomes. Genome Biol. 14, R22 (doi:10.1186/gb-2013-14-3-r22) 23497673 10.1186/gb-2013-14-3-r22 PMC3663115

[22] Rokas A, Holland PW 2000 Rare genomic changes as a tool for phylogenetics. Trends Ecol. Evol. 15, 454–459. (doi:10.1016/S0169-5347(00)01967-4) 11050348 10.1016/s0169-5347(00)01967-4

[23] Kriegs JO, Churakov G, Kiefmann M, Jordan U, Brosius J, Schmitz J 2006 Retroposed elements as archives for the evolutionary history of placental mammals. PLoS Biol. 4, e91 (doi:10.1371/journal.pbio.0040091) 16515367 10.1371/journal.pbio.0040091 PMC1395351

[24] Nishihara H, Maruyama S, Okada N 2009 Retroposon analysis and recent geological data suggest near-simultaneous divergence of the three superorders of mammals. Proc. Natl Acad. Sci. USA 106, 5235–5240. (doi:10.1073/pnas.0809297106) 19286970 10.1073/pnas.0809297106 PMC2655268

[25] Churakov G, Kriegs JO, Baertsch R, Zemann A, Brosius J, Schmitz J 2009 Mosaic retroposon insertion patterns in placental mammals. Genome Res. 19, 868–875. (doi:10.1101/gr.090647.108) 19261842 10.1101/gr.090647.108 PMC2675975

[26] Carelli FN, Hayakawa T, Go Y, Imai H, Warnefors M, Kaessmann H 2016 The life history of retrocopies illuminates the evolution of new mammalian genes. Genome Res. 26, 301–314. (doi:10.1101/gr.198473.115) 26728716 10.1101/gr.198473.115 PMC4772013

[27] Rohozinski J 2017 Data from: Lineage independent retrotransposition of UTP14 associated with male fertility has occurred multiple times throughout mammalian evolution Dryad Digital Repository. (http://dx.doi.org/10.5061/dryad.250dt) 10.1098/rsos.171049 PMC5750009 29308242

