# Supplementary material for: Lineage-independent retrotransposition of UTP14 associated with male fertility has occurred multiple times throughout mammalian evolution
Source: R Soc Open Sci. 2017 Dec 20;4(12):171049. doi: 10.1098/rsos.171049 (PMC5750009; doi:10.1098/rsos.171049)
Supplement: Fig S1. Divergence of UTP14 retrogene peptides encoded within the genomes of African elephant and aardvark. [file rsos171049supp1.docx]

Figure S1.

**Elephant MSATGAAESLLTLSHQEELADLPKDCPWSTSEDEGDSDRERKHQKLLEAISSLDGKNRRK 60**

**MSA GAAE+LL L QEEL DLPKD STSEDEGDSDRE KH+KLLEAI SLDGK+R +**

**Aardvark MSAAGAAENLLALCQQEELVDLPKDYTVSTSEDEGDSDREWKHRKLLEAIRSLDGKSRWE 60**

**Elephant LAERSEASWDVSEFSVGCEGTGEQLVLSDLLQPIKTSSSLATVKKQLKGVQAKKTVELPL 120**

**AERSEA ++SEF+V EG G++LVL+DLLQP K SSSLA V+KQLK VQ+K TVELPL**

**Aardvark SAERSEAHLNMSEFNVSSEGIGDKLVLADLLQPSKASSSLAAVRKQLKRVQSK-TVELPL 119**

**Elephant DQEEAAQVHREVAFSTTSQALSKWDPVVLKNRNAEQLVFPLKQQQLDFAPIEHVLGGWKA 180**

**EE A++HREVAFS TSQALSKWDP+V+KNR AEQL FPL+++ FAPIEHVL GWKA**

**Aardvark CAEEVARIHREVAFSQTSQALSKWDPIVVKNRKAEQLAFPLQKEPGAFAPIEHVLRGWKA 179**

**Elephant RSPLEQEIFNVLHKNKQPVIDPLLTPVEKASLKATSLEEAQMRQAELQKARALQSYYEAK 240**

**R+ LE+EI+NVLHKNKQPV+DPLLTP EKASLKA SLEEA++R+AELQ+ARALQSYYEA+**

**Aardvark RTALEEEIYNVLHKNKQPVLDPLLTPAEKASLKAMSLEEAKLRRAELQRARALQSYYEAR 239**

**Elephant ARREKKIKSKKYHKVVKKGKAKIALKDFETLQKASPTTALEELEKIEKARMVERMSLKHQ 300**

**ARREK+IKS+KY + VKKGKAK AL++ E L K PT AL E KARM+ER+SL+HQ**

**Aardvark ARREKRIKSRKYRRAVKKGKAKKALRELEALGKVCPTAALGR-ETTGKARMMERLSLRHQ 298**

**Elephant NKGKWAKSKAIMAKYDPEARQAMHEQLAKNKELTQKLQAPSESEEEEGAVEEDGLFVPDV 360**

**+ GK AKS+A+ A+YDP ARQAM EQ AKNKEL QKLQ PSESEEEEG EE GL VPDV**

**Aardvark DNGKRAKSRAVRARYDPAARQAMQEQWAKNKELVQKLQEPSESEEEEGGAEEVGLLVPDV 358**

**Elephant VNEVQMDTHGPNPWMHTSCTSDVKEAEVLKDPEQLPEHLAFEDSEGEGEEGPVAEEAMLL 420**

**+ V ++THGPNPWMH SCTSD +EAE+ KDP QLP H + SE E EE PVAEE L**

**Aardvark SHRVPVNTHGPNPWMHRSCTSDAREAEIQKDPAQLPRHASCGGSEREEEESPVAEEEPL- 417**

**Elephant KESEERRCLRKRPELNQDTKPVGRQETQDPSSQEVLSELRALSQKLSKEDRQPRKQKANS 480**

**KE EER LRKR E QD +PVG+QETQDPSSQEVLSELRALS +L KE + RKQ+ +S**

**Aardvark KEFEERPSLRKRAEPGQDAEPVGQQETQDPSSQEVLSELRALSLQLGKESHRLRKQRVDS 477**

**Elephant AGAILPVQREEPAREEEEPLLPQRPERVQTPKEL---GKGGCSQNTELRRPVLQGQQMEK 537**

**GA+L VQ +E+EPLL QRPE V+ +EL G GC QNT R V +GQQ E+**

**Aardvark VGAVLLVQ------DEKEPLLLQRPEGVRPLEELEALGLRGCLQNTGPPRCVFRGQQTER 531**

**Elephant NANNQPDAPKKKKRKEQMIDLQNLLTTKSPSRESLAVPTTIDKLEGEEERDQKQMIKEAF 597**

**N + QPDAP+KKKR +MIDLQ+LLTT SP + S+A PT I++LEGEEERD++QMIK+AF**

**Aardvark NPDTQPDAPRKKKRV-RMIDLQDLLTTHSPGK-SMAGPTVIEELEGEEERDERQMIKDAF 589**

**Elephant AGDDVIRDFLKEKREAVEASKPKDVDLTLPGWGEWGGVGLQPRAKKRRCFLMKAPEGPPR 657**

**AGDDVIRDFL EKR AVEA+KPKDVDL LPGWGEWGGVGLQP A+KRR FLMKAPE PPR**

**Aardvark AGDDVIRDFLDEKRAAVEANKPKDVDLMLPGWGEWGGVGLQPGARKRRRFLMKAPERPPR 649**

**Elephant KDENLPNVIISEERNIHAAAHQVHVLPYPFSHHQQFERTIRTPIGSMWNTQRAFHKLTTP 717**

**KDENLPNV+ISE+RNI AA HQVH LP+PF+H QQF+RTI+TP GS WNTQRAF KLT P**

**Aardvark KDENLPNVVISEKRNICAAVHQVHALPFPFTHRQQFKRTIQTPAGSTWNTQRAFQKLTMP 709**

**Elephant KVVTKPGHIIKPIEAEDVGYRSSSRLDLSVVQRNPKRLSIHHKRPLKKNSVG 769**

**+VVTKPGHII P+ AEDVGYRSSSR DLSVVQRNPK LSI HK+**

**Aardvark RVVTKPGHIITPLSAEDVGYRSSSRSDLSVVQRNPKPLSIRHKK 753**
