# Supplementary figures and images for: Lineage-independent retrotransposition of UTP14 associated with male fertility has occurred multiple times throughout mammalian evolution
Source: R Soc Open Sci. 2017 Dec 20;4(12):171049. doi: 10.1098/rsos.171049 (PMC5750009; doi:10.1098/rsos.171049)

## Slide 1
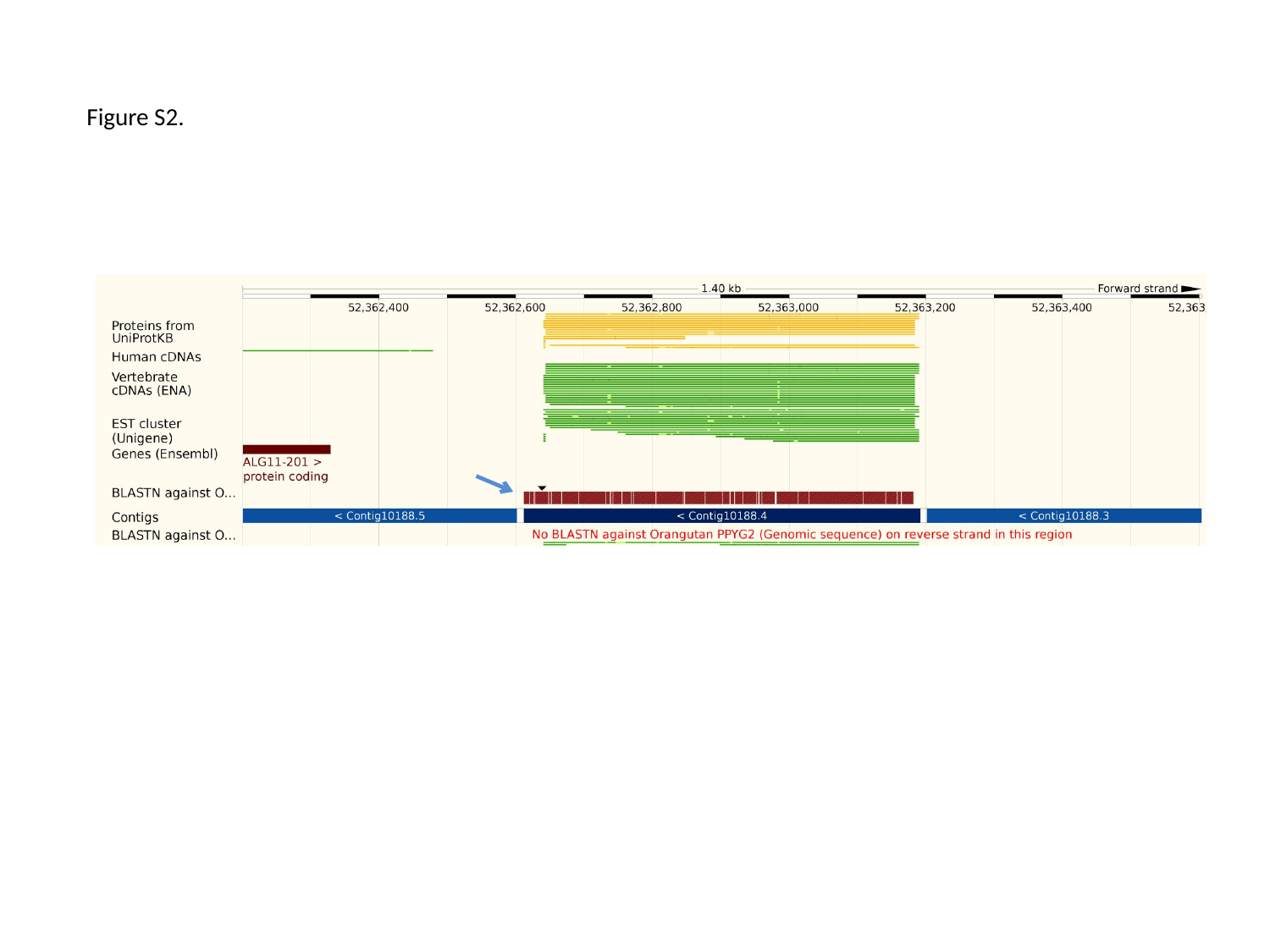

Figure S2.

Supplement: Fig S2. Half of orangutan UTP14C has been deleted without impacting the function of its host gene ALG11. [file rsos171049supp2.pptx]

## Slide 1
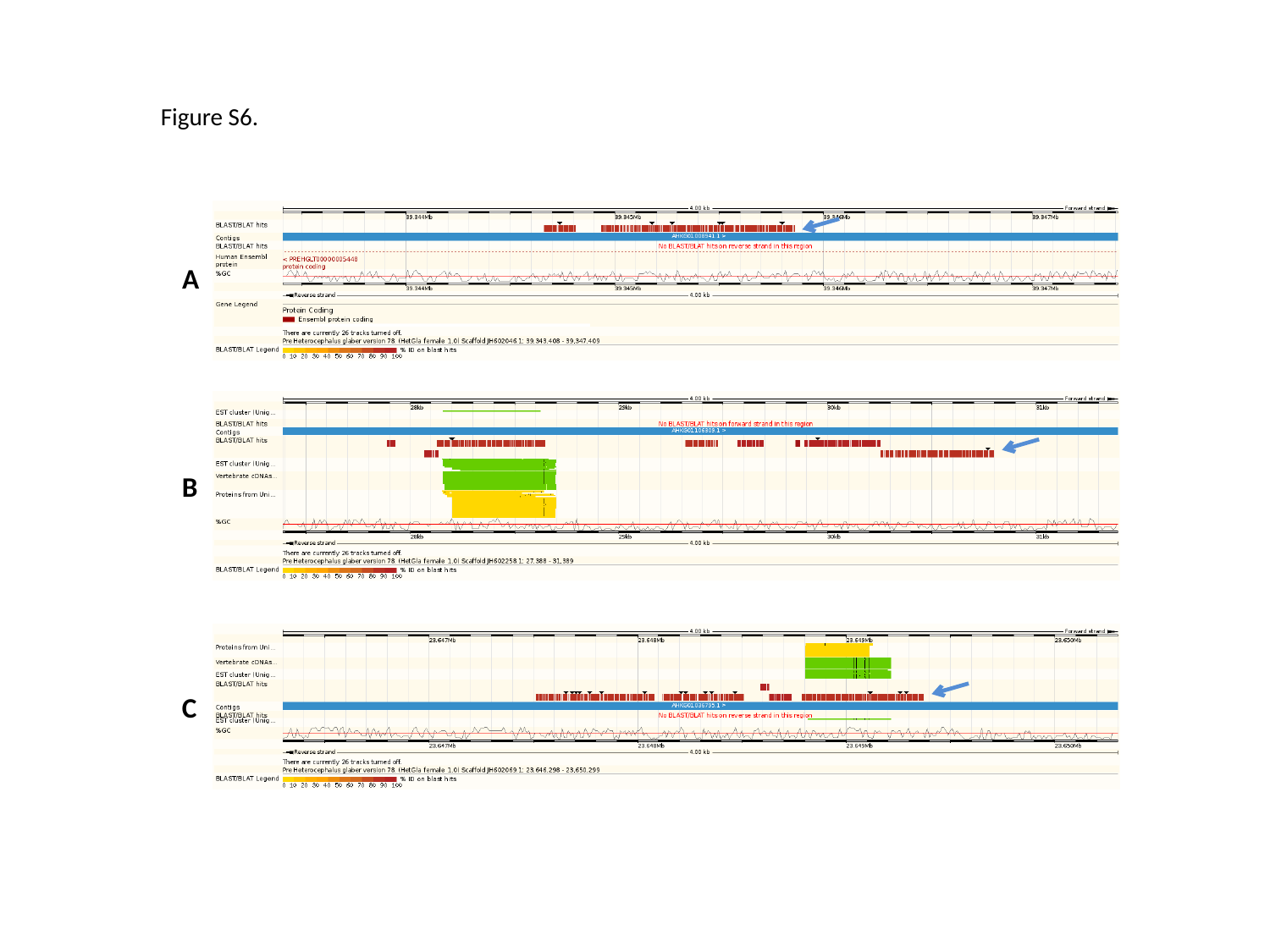

Figure S6.
A
B
C

Supplement: Fig S6. In addition to a functional copy of a UTP14 retrogene the naked mole rat genome caries three nonfunctional copies. [file rsos171049supp6.pptx]
