# Supplementary material for: Lineage-independent retrotransposition of UTP14 associated with male fertility has occurred multiple times throughout mammalian evolution
Source: R Soc Open Sci. 2017 Dec 20;4(12):171049. doi: 10.1098/rsos.171049 (PMC5750009; doi:10.1098/rsos.171049)
Supplement: Fig S3. Alignment of the terminal exon and flanking 3' sequence of ALG11 from the bush baby, human, marmoset and squirrel monkey identifies potential points of retrogene insertion and deletion. [file rsos171049supp3.docx]

Figure S3.

**Bush Baby GAGTTGTGGAGTGTATGGCAGCTGGCACAGTCATCCTTGCACATAATTCA**

**Human GAGTTGTGGAGTGTATGGCAGCTGGCACAATTATCCTTGCACACAATTCG**

**Marmoset GAGTTGTGGAGTGTATGGCAGCTGGCACAATTATCCTTGCACACAATTCA**

**Squirrel Monkey GAGTTGTGGAGTGTATGGCAGCTGGCACAATTATCCTTGCACACAATTCA**

******************************* * *********** *******

**Bush Baby GGGGGCCCGAAGCTTGACATTGTTGTTCCTCATGAAGGAGAGATAACTGG**

**Human GGGGGCCCAAAGCTTGACATTGTGGTTCCTCACGAAGGAGATATAACTGG**

**Marmoset GGGGGCCCAAAGCTTGACATTGTTGTTCCTCACAAAGGAGATATTACTGG**

**Squirrel Monkey GGGGGCCCGAAGCTTGACATTGTTGTTCCTCACGAAGGAGATATAACTGG**

********** ************** ******** ******* ** *******

**Bush Baby ATTTCTGGCTGAGAGTGAAGAAGGCTATGCTGAGACTATGGCTCGTATTC**

**Human CTTTCTGGCTGAGAGTGAAGAAGACTATGCTGAAACTATCGCTCACATTC**

**Marmoset CTTTCTGGCTGAGAGTGAAGAAGGCTATGCTGAAACTATGGCTCACATTC**

**Squirrel Monkey CTTTCTGGCTGAGAGTGAAGAAGGCTATGCTGAAACTATGGCTCACATTC**

************************ ********* ***** **** ******

**Bush Baby TTTCCATGTCTGCAGAAGAGAGATTCCAAATCAGAAAAAATGCTCGCGCG**

**Human TTTCCATGTCTGCAGAAAAGAGACTCCAAATCAGAAAAAGTGCTCGTGCA**

**Marmoset TTTCCATGTCTGCAGAAAAGAGACTCCAAATCAGAAAAAGTGCCCGTGCA**

**Squirrel Monkey TTTCCATGTCTGCAGAAAAGAGACTCCAAATCAGAAAAAGTGCCCGTGCA**

******************* ***** *************** *** ** ****

**Bush Baby TCTGTAAGCAGGTTCTCTGATCAGGAGTTTGAAGTGACATTCCTGTTATC**

**Human TCTGTAAGCAGATTCTCTGATCAGGAATTTGAAGTGACATTCCTATCATC**

**Marmoset TCTGTAAGCAGATTCTCTGATCAGGAATTTGAAGTGGCATTTCTATCATC**

**Squirrel Monkey TCTGTAAGCAGATTCTCTGATCAGGAATTTGAAGTGGCATTTCTATCATC**

************* ************** ********* **** ** * *****

**Bush Baby TGTGGAAAAGTTATTTAAATAATGCCATATATGTACAAATTAAAGGTATT**

**Human TGTGGAAAAGTTATTTAAGTAATGCCATATCTGTA-AAATTAAAGATATT**

**Marmoset TGTGGAAAAGTTATTTAAGTAATCTCGTATCTGTA-ACATTAGAGATATT**

**Squirrel Monkey TGTGGAAAAGTTACTTAAGTAATGCCATATCTATA-AAATTAAAGATATT**

*************** **** **** * *** * ** * **** ** ******

**▼**

**Bush Baby TTATATAAAATTATTAAACCCCTTCATATGTAAATATTTTTCTAAACCCG**

**Human TTATATAAACTGGTTAAACACCTTCATATGTAAATATTTTTCTAAATTCA**

**Marmoset TTATATAAAATGGTTAAAAACCTTCATATGTAAATATTTTTCTAAATTCA**

**Squirrel Monkey TTATATAAAATGGTTAAAAACCTTCACATGTAAATATTTTTCTAAATTCA**

*********** * ***** ****** ******************* ***

**Bush Baby TTCCCTGTTGTAATAAAGTCAGCCTAT-GCAGTATTC--GTAGCAGTATG**

**Human ATCTCATTTGTCA---AATCATTTTACTTTAGAAAACAG---ACAAAATT**

**Marmoset ATCTCATTTGTCA---AATCACTTTACTTTAGGAAACAAGAAAGAAAATT**

**Squirrel Monkey ATCTCATTTGTCA---AATCACTTTACTTTAGAAAACAGGAAAGAAAATT**

**** * **** * * *** ** ** * * * ****

**▼**

**Bush Baby TACATAGGCAAGGTATTTATTTCACTGTAAAAAGGCAAAAT-CAGATTAG**

**Human TCCTTTTAGA----ATAAAAGGAAGTGTTGAAAAGAAAATGGATGACTAG**

**Marmoset TCCTTTTATA----ATAAAAGGAAGTGTTGAAAGGAAAATAGATGACCAG**

**Squirrel Monkey TCCTTTTAGA----ATAAAAGGAAGTGTTGAAAGGAATTGGTATAAATTT**

*** * * * ** * * *** *** * * ***

**Bush Baby TATGTGGCTCTAAGCTCT------------AAAAA----ATATGAAT-CT**

**Human CCTTCGGCTTCCATTCTTGGTATA--CATGAGAGA----GGCTGGCTGCT**

**Marmoset ACTTCGGCTTTCATTCTTGGTCCA--CATGAGAGA----AGCTGGCTGCT**

**Squirrel Monkey AATGCAACCTTTTTTTTTTTTTTTTTTTTTTGAGACGGAGTTTCGCTC--**

*** * * * * * ***

**Bush Baby GAAAGAAA--AAAATTAAGTCAT---GA--AATGT-GC--TATAATCCAT**

**Human GAGATGAATGTGAACCAGGTTGCAGAGA---ATCTGGC--TTTGAGCCAC**

**Marmoset GAAATGAGTGCGAACCAGGTTGTGGAGAGCCTTCTGGC--TTTGAGCCAA**

**Squirrel Monkey -------TTGTTACCCAGGTTGGAGTG--CAATGGCGCGATCTCGGCTCA**

*** * ** * * ** * * ***

**Bush Baby ATTTTT----AAA--ATTTCATTT-GTCAATT-----CACTTTTAAG--A**

**Human CAGGAAGA----ACTAGTGGATTT-GCCAAAAAACTACCC-CTTGAGTGA**

**Marmoset CAGGAAGA----ACTGGTGGATTT-GCCAAAAGACCACTC-CTTGAGTGA**

**Squirrel Monkey CCGCAACCTCCGCCTCCTGGGTTCAGGCAATT-----CTC--CTGCCTCA**

*** ** * *** * * * ***

**Bush Baby AACAGGAAAGAAAT-----TTTTCTTTTAGGA---AAAAAATAA-GTGTT**

**Human AAATGAAGATGAGGGGGACAGTGA-TGGAGAGAGAAAGCATCAAAAGCTT**

**Marmoset GAGTGAAGATGAGGGGGACAGTGA-TGGAGAGAGAAAGCATCAAAAGCTT**

**Squirrel Monkey GCCTCCTGAGTAGC-----TGGGATTACAGGC---ACGCACCACCATGCC**

*** * * ** * * ***

**Bush Baby C---TGTCAGCATCTGTTTTTGCTTAAAAAACAAAAGGGAAGAAA-GAAA**

**Human C---TGGAAGCAATCAT-TTCCCTTGATG--GAAA-GAATAGGCG-GAAA**

**Marmoset C---TGGAAGCAATCCA-TTCCCTTGATG--GAAA-GAATAGACA-GAAA**

**Squirrel Monkey CAGCTGATTTTTTTGTA-TT--TTTAGTA--GAGACGGGGTTTCACCATG**

*** ** ** ** * * * ***

**Bush Baby ATG-TTGATA-AGAAA-ATGAATGACTGATAATTGGCACTTTAGAATAAT**

**Human TTGGCTGAGA-GGTCTGAGGCTAGTCTGAAA-GTGTCAGAGTTCAGTGTC**

**Marmoset TTGACTGAGA-GGTCTGAGGCTGGAATGAAG-GTGTCAGAGTTCAATGTC**

**Squirrel Monkey TTGACCAGGATGGTCTCGATCT--CCTGACC-TTGTGATCCACCCGCCTC**

**** * * *** ** ***

**Bush Baby TGT-TCTGACATTTTTGGAACACAGA---AAAATTCTGATGCGTT----T**

**Human AGT-TCTGAAGGATCAGGAGAAAAGCTGGGCCTTGCAGATCTGCT----T**

**Marmoset AGT-TCTGAAGGATCAGGAGAAAAGCTGGTCCTTGCAGATCTGCT----T**

**Squirrel Monkey AGCCTCCCAAA-----------GTGCTGGGA-TTACAGGCTTGAGCCACC**

*** ** * * * * * ***

**Bush Baby AAATT----CTAAACAATATTGTAGATGACCCTTTGTAATATACCTAATG**

**Human GAGCCCG-TTAAAACTTCATCTTCTTTGG-CCACTGTAAAAAAGCAACTG**

**Marmoset GAGCCTG-TTAAAACTTCATCTTTTTTGG-CCACTGTGAAAAAGCAACTG**

**Squirrel Monkey GCGCCTGGCCTAAATTTAATGCAACTT---CTATCCTGAAAAACTAGCAT**

***** ** * * * * * ***

**Bush Baby ATTTTCTTGAAAGAAACTTTAAATAAGCTTATTTAATCA--TGTTCCCAA**

**Human AATA----GAGTCAAA--TCAAAGAAGGTGGTGGAGTTACCTCTTAACAA**

**Marmoset AACA----GAGTCAAA--TCAAAGAAGGTAGTGGAGTTACCTCTTAACAA**

**Squirrel Monkey GGAA----AAGTGAAT--ACATAT--GTGGGCAGACATGGCTATAAAGAC**

*** ** * * * * * * ***

**Bush Baby AGCATACAGTCAAGAGAC-A-AGACTGACT-GATGTTTTCTGAGT-GTAT**

**Human AGAAAAAATTGAACAGATC---CACAGAGAAGTAGCATTC-----AGTAA**

**Marmoset AGAAAAGATTGAACAGATC---CACAGAGAAGCAGCATTC-----GGTAA**

**Squirrel Monkey CT-AAAACTTCTGCATTTTCTGCCTATATAATCAATCCTTTC--TAGTAC**

*** * * * * * *****

**Bush Baby AAG----CATGTGA-GGGCAAAGTACTG-CCAGCAGTCCTTTCAAT--AA**

**Human AACCTCACAGGTC-CTCTCCAAATGGGACCCTATCATCC--TGAAG--AA**

**Marmoset AACCTCACAAATC-CTCTCCAAATGGGACCCTGTCATCC--TGAAG--AA**

**Squirrel Monkey AGA--GACTTGACCCCTTCCATAG---CCTGATTCATCT--TTGTGCTAA**

*** * * * ** * ****

**Bush Baby TTAGGAAAC-ATTTGTGAAAATGGTTTTTGT------GTAGGAC-TA-TC**

**Human CCAGCAGGC-A---GAGCAGCTGGTTTTTCCCCTGGGGAAGGAG-CAGCC**

**Marmoset CCGGCAGGC-A---GAGCAGCTGGCTTTTCCCCTAGGGAAGGAG-CAGCC**

**Squirrel Monkey GGGGCAAATGA---AAGGATATA-TTATT---CTTTTGCAGTCTCTTCTC**

*** * * * * * * ** * ** ***

**Bush Baby AGCA----CTTTCATGTGAATCT---CTCTTTATCTCTTAGCT--CCACA**

**Human AGCCATTGCTCCCAT-TGAACATGCGCTCAGTGGCTGGAAGGCAAGAACT**

**Marmoset AGCCATTGCTCCCAT-TGAACATGTGCTCAGTGGCTGGAAGGCAAGAACT**

**Squirrel Monkey AGTCATT-CACCAATCTGGCCA---GCTTATCTACTCCCAATTAGGTTGT**

**** * ** ** ** ** ***

**Bush Baby TC-TCA-GAGGCTGGAAGTTACCTTCAAGTCATTCAAGAGAA------CA**

**Human CC-CCT-GGAGCAGGAAATTT--TTAACCTCCTCCATAAGAA------CA**

**Marmoset CC-CCT-GGAGCAGGAAATTT--TTACCCTCCTCCACAAGAA------CA**

**Squirrel Monkey TGATATATCTGCAGGCCATCT--GTCATCGGATCAAAAAGTAGCAAAACA**

**** ** * * * * ** * ****

**Bush Baby T-CAGATGGTTTTTCTGACATGAGCTCCATCTGCAAAATGCATTTTGAAA**

**Human AGCAGCC------AGTGACAG-A--TCC-TTTACTGACTC-CCATGGAAA**

**Marmoset AGCAGCC------AGTGACAG-A--CCC-TTTACTGACTC-CCGTGGAAA**

**Squirrel Monkey GAGGGTC------AGTCACAG-G--AGG-TT--CTGACACACCATTGTAA**

*** * *** * * * * * ****

**Bush Baby AGAACATTGGACAAGCAGTT---------------AGCATTCCTGAGCCT**

**Human AGGCCTCTCTCCAAGCCATG---------------AGCCTGGAAGAGGCA**

**Marmoset AGGCCTCTCTCCAAGCCATG---------------AACCTGGAAGAGGCA**

**Squirrel Monkey ----CTTTGTGTTAGAGATGATGCCATTTAGAAAAAGACTGGTAGAAA--**

*** * ** * * * ****

**Bush Baby ACCCTTAGCTTCATCATGACAAATGTCTGACCTTTTGACACTAAAGCTGC**

**Human -----AAGATGCACCGAG--------------------------------**

**Marmoset -----AAGATGCACCGAG--------------------------------**

**Squirrel Monkey --------TTGGAATGAAAGGAACCC------------------------**

*** ***

**Bush Baby TCCTTAATCTTTCTGAGGCTCAG-AATCTTC--ATGT--ATAATCTGGTG**

**Human ----CAGAGCTTCAGAGGGCTCGGGCTCTGCAGTCC--TACTATGAGGCC**

**Marmoset ----CAGAGCTTCAGAGGGCCCGGGCTCTGCTGTCC--TACTATGAGGCC**

**Squirrel Monkey --TACAGATTTTCT--AGCCCAGTTCTCTCTTATTTTCAGCTTTACAGAC**

*** *** * * *** * ***

**Bush Baby -ATG------------T-------TAATCTAT--ATCCTTACTACCTTCT**

**Human AAGGCTCGAAAAGAGAAGAAAATCAAAAGTAAAAAGTATCACAAAGTCGT**

**Marmoset AAGGCTCAAAGAGAGAAGAAAATAAAAAATAAAAAGTATCACAAAGTTGT**

**Squirrel Monkey AAGA------------AC-AATTTAAATCTAAAGAATTTAGCAGACTCCT**

*** ** ** * * * * ***

**Bush Baby ATAGTTGTG-ATACTACT-----TTGAAAACTATAAACGTTATTTTCTAT**

**Human GAAGAAAGG-AAAG-GCC-----AAGAAAGCCTTAAAAGA-G-TTTGAGC**

**Marmoset AAAGAAAAG-AAAG-GCC-----AAGAGAGCCTTAAAAGA-C-TTTGAAC**

**Squirrel Monkey TCAGTGACACAAA--GCTGTTTAATGAAAGAATCAAAATT-ATAACCTGG**

**** * * * ** * *****

**Bush Baby AA-AAGTAACTATATATATAGGATGGACATAAAGTT-TGTGTGC**

**Human AGCTACAGAAGGTTAATCCAA--CT---GTGGCACTGGAAGAAA**

**Marmoset AACTGCAGAAGGTTAATCCGA--CT---GCAGCACTGGAAGAAA**

**Squirrel Monkey ATATTCTGACTCCTGGCCCAG--TG---CTTTTTCT-GACTTTG**
