# Supplementary material for: Lineage-independent retrotransposition of UTP14 associated with male fertility has occurred multiple times throughout mammalian evolution
Source: R Soc Open Sci. 2017 Dec 20;4(12):171049. doi: 10.1098/rsos.171049 (PMC5750009; doi:10.1098/rsos.171049)
Supplement: Fig S4. Alignment of squirrel monkey UTP14A and the retrogenes UTP14D and E reveals limited sequence divergence. [file rsos171049supp4.docx]

Figure S4.

**UTP14D ATGACTGCGAACCGGCTCACGGAGAGCCTTCTGGCTTTGAGCCAACAGGAAGAACTAGTG**

**UTP14A ATGACTGCGAACCGGCTCACGGAGAGCCTTCTGGCTTTGAGCCAACAGGAAGAACTAGTG**

**UTP14E ATGACTGCGAACCGGCTtACGGAG----------------------AGGAAGAACTAGTG**

**UTP14D GATTTGCCAAAAGACTACCTCTTGAGTGAGAGTGAAGATGAGGGGGACGGTGATGGAGAG**

**UTP14A GATTTGCCAAAAGACTACCTCTTGAGTGAGAGTGAAGATGAGGGGGACGGTGATGGAGAG**

**UTP14E GATTTGCCAAAAGACTACCTCTTGAGTGAGAGTGAAGATGAGGGGGACGGTGATGGAGAG**

**UTP14D AtAAAGCATCAGAAgCTTCTGGAAGCAATCAGTTCCCTTGATaGAAAGAATAGGCGGAAA**

**UTP14A AGAAAGCATCAGAAgCTTCTGGAAGCAATCAGTTCCCTTGATGGAAAGAATAGGCGGAAA**

**UTP14E AGAAAGCATCAGAAcCTTCTGGAAGCAATCAGTTCCCTTGATGGAAAGAATAGGCaGAAg**

**UTP14D TcaGCTGAGAGGTCtGAGGCTAGTCTGAAGGTATCAGAGTTCAATGTCAGTTCTGAAGGA**

**UTP14A TTGGCTGAGAGGTCgGAGGCTAGTCTGAAGGTATCAGAGTTCAATGTCAGTTCTGAAGGA**

**UTP14E TTGGCTGAGAGGTCcGAGGCTAGTCTGAAGGTATCAGAGTTCAATGTCAGTTCTGAAGGA**

**UTP14D TCAGGAGAAAAGCTGGTCCTTGCAGATCTGCTTGAGCCTGTTAAAACTTCATCTTCTTTG**

**UTP14A TCAGGAGAAAAGCTGGTCCTTGCAGATCTGCTTGAGCCTGTTAAAACTTCATCTTCTTTG**

**UTP14E TCAGGAGAAAAGCTGGTCCTTGCAGATCTGCTTGAGCCTGTTAAAACTTCATCTTCTTTG**

**UTP14D GCTACTGTGAAAAAGCAACTGAATAGAGTCAAATCAAAGAAGACCGTGGAGTTACCTCTT**

**UTP14A GCTACTGTGAAAAAGCAACTGAATAGAGTCAAATCAAAGAAGACCGTGGAGTTACCTCTT**

**UTP14E GCTACTGTGAAAAAGCAACTGAATAGAGTCAAATCAAAGAAGACCGTaGAGTTACCTCTT**

**UTP14D AACAAAGAAGAGATTGAACGGATCCACAGAGAAGTAGCATTCAATAAAACCTCACAAGTC**

**UTP14A AACAAAGAAGAGATTGAACGGATCCACAGAGAAGTAGCATTCAATAAAACCTCACAAGTC**

**UTP14E AACAAAGAAGAGATTGAACGGATCCACAGAGAAGTAGCATTCAATAAAACCTCACAAGTC**

**UTP14D CTCTCCAAATGGGACtCTATTGTCCTAAAGAACCGACAGGCAGAGCAGCTGGTTTTTCCC**

**UTP14A CTCTCCAAATGGGACCCTATTGTCCTAAAGAACCGACAGGCAGAGCAGCTGGTTTTTCCC**

**UTP14E CTCTCCAAATGGGACCCTATcGTCCTAAAGAACCGACAGGCAGAGCAGCTGGTTTTTCtC**

**UTP14D CTGGgGAAGGAGGAGCCAATCATTGCTCCCATTGAACATGTGCTCAGTAGCTGGAAGGCA**

**UTP14A CTGGAGAAGGAGGAGCCAATCATTGCTCCCATTGAACATGTGCTCAGTAGCTGGAAGGCA**

**UTP14E CTGGAGAAGGAGGAGCCAATCATTGCTCCCATTGAACATGTGCTCAGTAGCTGGAAGGCA**

**UTP14D AGAACTCCtCTGGAGCAAGAAATTTTCAACCTCCTCCATAAGAACAAGCAGCCAGTGACA**

**UTP14A AGAACTCCCCTGGAGCAAGAAATTTTCAACCTCCTCCgTAAGAACAAGCAGCCAGTGACA**

**UTP14E AGAACTCCCCTGGAGCAAGAAATTTTCAACCTCCTCCATAAGAACAAGCAGCCAGTGACA**

**UTP14D GACCCTTTACTGACTCCCGTAGAAAAGGCCTCTCTCCAAGCCATGAGCCTGGAAGAGGCA**

**UTP14A GACCCTTTACTaACTCCCGTAGAAAAGGCCTCTCTCCAAGCCATGAGCCTGGAAGAGGCA**

**UTP14E GACCCTTTACTGgCTCCCGTAGAAAAGGCCTCTCTCCAAGCCATGAGCCTGGAAGAGGCA**

**UTP14D AAGaTGCGCCGAGCAGAGCTTCAGAGGGCCCGGGCTCTGCAGTCCTACTATGAGGCCAAG**

**UTP14A AAGGTGCGCCGAGCAGAGCTTCAGAGGGCCCGGGCTCTGCAGTCCTACTATGAGGCCAAa**

**UTP14E AAGGTGCGCCGAGCAGAGCTTCAGAGGGCCCGGGCTCTGCAGTCCTACTATGAGGCCAAG**

**UTP14D GCTCGAAGAGAGAAGAAAATTAAAAGTAAAAAGTATCACAAAGTCaTGAAGAAAGGAAAG**

**UTP14A GCTCGAAGAGAGAAGAAAATTAAAAGTAAAAAGTATCACAAAGTCGTGAAGAAAGGAAAG**

**UTP14E GCTtGAAGAGAGAAGAAAATTAAAAGTAAAAAGTATCACAAAGTCGTaAAGAAAGGAAAG**

**UTP14D GCCAAGAAAGCCCTAAAAGAGTTTGAGCAGCTGCGGAAGtTTAATCCAGCTGCAGCACTG**

**UTP14A GCCAAGAAAGCCCTAAAAGAGTTTGAGCAGCTGCGGAAGGTTAATCCAGCTGCAGCAtTG**

**UTP14E GCCAAGAAAGCCCTAAAAGAGTTTGAGCAGCTGCGGAAGGTTAATCCAGCTGCAGCACTG**

**UTP14D GAAGAACTGGAAAAAATTGAAAAGGCCAGAATGATGGAAAGAATGAGCCTTAAGCACCAA**

**UTP14A GAAGAACTGGAAAAAATTGAAAAGGCCAGAATGATGGAAAGAATGAGCCTTAAGCACCAA**

**UTP14E GAAGAACTGGAAAAAATTGAAAAGGCCAGAATGATGGAAAGAATGAGCCTTAAGCACCAA**

**UTP14D AACAGTGGGAAATGGGCCAAATCAAAGGCAATTATGGCCAAATATGACCTGGAGGCTCGC**

**UTP14A AACAGTGGGAAATGGGCCAAATCAAAGGCAATTATGGCCAAATATGACCTGGAGGCTCGC**

**UTP14E AACAGTGGGAAATGGGCCAAATCAAAGGCAATTATGGCCAAATATGACCTGGAGGCTCGC**

**UTP14D CAgGCTATGCAGGAACAGTTGGCCAAGAACAAAGAACTGgCACAGAAACTCgAGGTAGCC**

**UTP14A CAAGCTATGCAGGAACAGTTGGCCAAGAACAAAGAACTGACACAGAAACTCAAGGTAGCC**

**UTP14E CAAGCTATGCAGGAACAGTTGGCCAAGAACAAAGAACTGACACAGAAACTCAAGGTAGCC**

**UTP14D TCTGAGAGTGAGGAGGAGGAGGGAGGCGCAGAAGTGGAAGAACTCCTTGTCCCTGATGCA**

**UTP14A TCTGAGAGTGAGGAGGAGGAGGGAGGCGCAGAAGTGGAAGAACTCCTTGTCCCTGATGCA**

**UTP14E TCTGAGAGTGAGGAGGAGGAGGGAGGCGCAGAAGTGGAAGAACTCCTTGTCCCTGATGCA**

**UTP14D GTGAATGAAGTGCAGATGAATGTGGACGGACCgAATCCCTGGATGCTCAGGAGCTGCgCC**

**UTP14A GTGAATGAAGTGCAGATGAATGTGGACGGACCAAATCCCTGGATGCTCAGGAGCTGCACC**

**UTP14E GTGAATGAAGTGCAGATGAATGTGGACGGACCAAATCCCTGGATGCTCAGGAGCTGCACC**

**UTP14D AGTGACACCAgAGAGGCTGCAACCGAGGAGGAGGACCCTGAGCAGCTGCCAGAGCCTGTG**

**UTP14A AGTGACACCAAAGAGGCTGCAACCGAGGAGGAGGACCCTGAGCAGCTGCCAGAGCCTGTG**

**UTP14E AGTGACACCAAAGAGGCTGCAACCGAGGAGGAGGACCCTGAGCAGCTGCCAGAGCCTGTG**

**UTP14D GCcCACGAAGTTTCTGAAAGTGAGGGAGAAGAAAGACCAGTGGCAGAAGAAGACATTTTG**

**UTP14A GCaCACGAAGTTTCTGAAAGTGAGGGAGAAGAAAGACCAGTGGCAGAAGAAGACATTTTG**

**UTP14E GCgCACGAAGTTTCTGAAAGTGAGGGAGAAGAAAGACCAGTGGCAGAAGAAGACATTTTG**

**UTP14D TTGAGAGAATTTGAGGAAAGGCGATCCCTTAGAaAAAGATCgGAGCTCAACCAGGATGCT**

**UTP14A TTGAGAGAATTTGAGGAAAGGCGATCCCTTAGACAAAGATCTGAGCTCAACCAGGATGCT**

**UTP14E TTGAGAGAATTTGAGGAAAGGCGATCCCTTAGACAAAGATCTGAGCTCAACCAGGATGCT**

**UTP14D GAGCCAGCAGACAGTCAAGAAACAAAAGATTCTAGTAGCCAGGAGGTGCTGTCCGAcTTG**

**UTP14A GAGCCAGCAGACAGTCAAGAAACAAAAGATTCTAGTAGCCAGGAGGTGCTGTCCGAATTG**

**UTP14E GAGCCAGCAGACAGTCAAGAAACAAAAGATTCTAGTAGCCAGGAGGTGCTGTCCGAATTG**

**UTP14D AGGGCACTGTCTCAGAAAcTGAAGGAcGACCATCAGTCCAGGAAGCAAAAAGCAAGTTCA**

**UTP14A AGGGCACTGTCgCAGAAATTGAAGGAAGACCATCAaTCCAGGAAGCAAAAAGCAAGTTCA**

**UTP14E AGGGCACTGTCTCAGAAATTGAAGGAAGACCATCAGTCCAGGAAGCAAAAAGCAAGTTCA**

**UTP14D GAGGgGACTGTTCCCCAGaTCCAGAGAGAGGAACCTGCCtCAGAAGAAGAGGAGCCCCTG**

**UTP14A GAGGCGACTGTTCCCCAGGTCCAGAGAGAGGAACCTGCCCCAGAAGAAGAGGAGCCCCTG**

**UTP14E GAGGCGACTGTTCCCCAGGTCCAGAGAGAGGAACCTGCCCCAGAAGAAGAGGAGCCCCTG**

**UTP14D TTGCTACAGAGGCCAGAAAGAGTACAGATGCTGGAAGAGCTAGAAAAcGAAGAATGTTTT**

**UTP14A TTGCTACAGAGGCCAGAAAGAGTACAGATGCTGGAAGAGCTAGAAAAAGAAGAATGTTTT**

**UTP14E TTGCTACAGAGGCCAGAAAGAGTACAGATGCTGGAAGAGCTAGAAAAAGAAGAATGTTTT**

**UTP14D CAAAATAAGGAGCTTTCCAGACCTGTGTTAGAAGGGCATTGGTCAGAGAAGACCCCAAAT**

**UTP14A CAAAATAAGGAGCTTTCCAGACCTGTGTTAGAAGGGCATTGGTCAGAGAAGACCCCAAAT**

**UTP14E CAAAATAAGGAGCTTTCCAGACCTGTGTTAGAAGGGCATTGGTCAGAGAAGACCCCAAAT**

**UTP14D AATCACCCTGATGCCCCTAAGGAGAAGAAAAAGAAGGAGCAAATGATTGACCTACAGAAC**

**UTP14A AATCACCCTGATGCCCCTAAGGAGAAGAAAAAGAAGGAGCAAATGATTGACCTACAGAAC**

**UTP14E AATCACCCTGATGCCCCTAAGGAGAAGAAAAAGAAGGAGCAAATGATTGACCTACAGAAC**

**UTP14D CTCCTAACCACACAGTCTCTCTCGGTGAAGTCTTTGGCAGTGCCCACAAcAGAGAcGCTG**

**UTP14A CTCCTAACCACACAGTCTCTCTCGGTGAAGTCTTTGGCAGTGCCCACAATAGAGATGCTG**

**UTP14E CTCCTAACCACACAGTCTCTCTCGGTGAAGTCTTTGGCAGTGCCCACAATAGAGATGCTG**

**UTP14D GAAGATGAcGTGGAGAGAAACCAAAGGCAGATGATAAAGGAAGCcTTCGCTGGGGATGAT**

**UTP14A GAAGATGAAGTGGAGAGAAACCAAAGGCAGATGATAAAGGAAGCTTTCGCTGGGGATGAT**

**UTP14E GAAGATGAAGTGGAGAGAAACCAAAGGCAGATGATAAAGGAAGCTTTCGCTGGGGATGAT**

**UTP14D GTCATCAGAGATTTCTTGAAAGAGAAGAGGGAgGCTGTGGAGGCCAGTAAGCCAAAGGAC**

**UTP14A GTCATCAGAGATTTCTTGAAAGAGAAGAGGGAAGCTGTGGAGGCCAGTAAGCCAAAGGAC**

**UTP14E GTCATCAGAGATTTCTTGAAAGAGAAGAGGGAAGCTGTGGAGGCCAGTAAGCCAAAGGAC**

**UTP14D GTgGACCTGACACTACCTGGCTGGGGCGAGTGGGGTGGTGTGGGCCTAAAGCCCAGTGCC**

**UTP14A GTAGACCTGACACTACCTGGCTGGGGCGAGTGGGGTGGTGTGGGCCTAAAGCCCAGTGCC**

**UTP14E aTAGACCTGACACTACCTGGCTGGGGCGAGTGGGGTGGTGTGGGCCTAAAGCCCAGTGCC**

**UTP14D AAGAAAAGACGCCGGTTTCTCATTAAAGCCCCTGAGGGTCCTCCAAGAAAAGATAAGAAT**

**UTP14A AAGAAAAGACGCCGGTTTCTCATTAAAGCCCCTGAGGGTCCTCCAAGAAAAGATAAGAAT**

**UTP14E AAGAAAAGACGCCaGTTTCTCATTAAAGCCCCTGAGGGTCCTCCAAGAAAAGATAAGAAT**

**UTP14D TTGCCAAATGTGATTATCAATGAGAAGCGCAACATCCACGCAGCGGCTCATCAGGTACGA**

**UTP14A TTGCCAAATGTGATTATCAATGAGAAGCGCAACATCCACGCAGCGGCTCATCAGGTACGA**

**UTP14E TTGCtAAATGTGATTATCAATGAGAAGCGCAACATCCACGCAGCaGCTCATCAGGTACGA**

**UTP14D GTGCTTCCATATCCATTTACCCACCATCGGCAATTTGAAAGGACCATCCAGACCCCTATA**

**UTP14A GTGCTTCCATATCCATTTACCCACCATCGGCAATTTGAAAGGACCATCCAGACCCCTATA**

**UTP14E GTGCTTCCATATCCATTTACCCACCATCGGCAATTTGAAAGGACCATCCAGACCCCTATA**

**UTP14D GGATCCACGTGGAACACCCAGAGAGCTTTCCAAAgGCTGACTACTCCTAAGGTCATCACC**

**UTP14A GGATCCACGTGGAACACCCAGAGAGCTTTCCAAAAGCTGACTACTCCTAAGGTCATCACC**

**UTP14E GGATCCACGTGGAACACCCAGAGAGCTTTCCAAAAGCTGACTACTCCTAAGGTCATCACC**

**UTP14D AAGCCAGGCCgTATCATTAAGCCCATAAAAGCAGAGGATGTGGGCTACCGGTCTTCCTCA**

**UTP14A AAGCCAGGCCATATCATTAAGCCCATAAAAGCAGAGGATGTGGGCTACCGGTCTTCCTCA**

**UTP14E AAGCCAGGCCATATCATTAAGCCCATAAAAGCAGAGGATGTGGGCTACCGGTCTTCCTCA**

**UTP14D AGGTCGGACCTCTCTGTCATACAGAGGAATCCAAAACGGGTCACCACgCGTCACAAAAAA**

**UTP14A AGGTCGGACCTCTCTGTCATACAGAGGAATCCAAAACGGGTCACCACACGTCACAAAAAA**

**UTP14E AGGTCGGACCTCTCTGTCATACAGAGGAATCCAAAACGGGTCACCACACGTCACAAAAAA**

**UTP14D CAGCTGAAGAAAAACTCTGTAGATTGA**

**UTP14A CAGCTGAAGAAAAACTCTGTAGATTGA**

**UTP14E CAGCTGAAGAAAAACTCTGTAGATTGA**
