# Supplementary material for: Lineage-independent retrotransposition of UTP14 associated with male fertility has occurred multiple times throughout mammalian evolution
Source: R Soc Open Sci. 2017 Dec 20;4(12):171049. doi: 10.1098/rsos.171049 (PMC5750009; doi:10.1098/rsos.171049)
Supplement: Fig S5. Translation of squirrel monkey UTP14E indicates that this gene may have only relatively recently been inactivated. [file rsos171049supp5.docx]

Figure S5.

**M T A N R L T E** R K N * W I C Q K T T S F1

* L R T G L R R G R T S G F A K R L P L F2

D C E P A Y G E **E E L V D L P K D Y L L** F3

1 ATGACTGCGAACCGGCTTACGGAGAGGAAGAACTAGTGGATTTGCCAAAAGACTACCTCT 60

**▲**

* V R V K M R G T V M E R E S I R T F W F1

E * E * R * G G R * W R E K A S E P S G F2

**S E S E D E G D G D G E R K H Q N L L E** F3

61 TGAGTGAGAGTGAAGATGAGGGGGACGGTGATGGAGAGAGAAAGCATCAGAACCTTCTGG 120

K Q S V P L M E R I G R S W L R G P R L F1

S N Q F P * W K E * A E V G * E V R G * F2

**A I S S L D G K N R Q K L A E R S E A S** F3

121 AAGCAATCAGTTCCCTTGATGGAAAGAATAGGCAGAAGTTGGCTGAGAGGTCCGAGGCTA 180

V * R Y Q S S M S V L K D Q E K S W S L F1

S E G I R V Q C Q F * R I R R K A G P C F2

**L K V S E F N V S S E G S G E K L V L A** F3

181 GTCTGAAGGTATCAGAGTTCAATGTCAGTTCTGAAGGATCAGGAGAAAAGCTGGTCCTTG 240

Q I C L S L L K L H L L W L L * K S N * F1

R S A * A C * N F I F F G Y C E K A T E F2

**D L L E P V K T S S S L A T V K K Q L N** F3

241 CAGATCTGCTTGAGCCTGTTAAAACTTCATCTTCTTTGGCTACTGTGAAAAAGCAACTGA 300

I E S N Q R R P * S Y L L T K K R L N G F1

* S Q I K E D R R V T S * Q R R D * T D F2

**R V K S K K T V E L P L N K E E I E R I** F3

301 ATAGAGTCAAATCAAAGAAGACCGTAGAGTTACCTCTTAACAAAGAAGAGATTGAACGGA 360

S T E K * H S I K P H K S S P N G T L S F1

P Q R S S I Q * N L T S P L Q M G P Y R F2

**H R E V A F N K T S Q V L S K W D P I V** F3

361 TCCACAGAGAAGTAGCATTCAATAAAACCTCACAAGTCCTCTCCAAATGGGACCCTATCG 420

S * R T D R Q S S W F F S W R R R S Q S F1

P K E P T G R A A G F S P G E G G A N H F2

**L K N R Q A E Q L V F L L E K E E P I I** F3

421 TCCTAAAGAACCGACAGGCAGAGCAGCTGGTTTTTCTCCTGGAGAAGGAGGAGCCAATCA 480

L L P L N M C S V A G R Q E L P W S K K F1

C S H * T C A Q * L E G K N S P G A R N F2

**A P I E H V L S S W K A R T P L E Q E I** F3

481 TTGCTCCCATTGAACATGTGCTCAGTAGCTGGAAGGCAAGAACTCCCCTGGAGCAAGAAA 540

F S T S S I R T S S Q * Q T L Y W L P * F1

F Q P P P * E Q A A S D R P F T G S R R F2

**F N L L H K N K Q P V T D P L L A P V E** F3

541 TTTTCAACCTCCTCCATAAGAACAAGCAGCCAGTGACAGACCCTTTACTGGCTCCCGTAG 600

K R P L S K P * A W K R Q R C A E Q S F F1

K G L S P S H E P G R G K G A P S R A S F2

**K A S L Q A M S L E E A K V R R A E L Q F3**

601 AAAAGGCCTCTCTCCAAGCCATGAGCCTGGAAGAGGCAAAGGTGCGCCGAGCAGAGCTTC 660

R G P G L C S P T M R P R L E E R R K L F1

E G P G S A V L L * G Q G L K R E E N * F2

**R A R A L Q S Y Y E A K A * R E K K I K** F3

661 AGAGGGCCCGGGCTCTGCAGTCCTACTATGAGGCCAAGGCTTGAAGAGAGAAGAAAATTA 720

K V K S I T K S * R K E R P R K P * K S F1

K * K V S Q S R K E R K G Q E S P K R V F2

**S K K Y H K V V K K G K A K K A L K E F** F3

721 AAAGTAAAAAGTATCACAAAGTCGTAAAGAAAGGAAAGGCCAAGAAAGCCCTAAAAGAGT 780

L S S C G R L I Q L Q H W K N W K K L K F1

* A A A E G * S S C S T G R T G K N * K F2

**E Q L R K V N P A A A L E E L E K I E K** F3

781 TTGAGCAGCTGCGGAAGGTTAATCCAGCTGCAGCACTGGAAGAACTGGAAAAAATTGAAA 840

R P E * W K E * A L S T K T V G N G P N F1

G Q N D G K N E P * A P K Q W E M G Q I F2

**A R M M E R M S L K H Q N S G K W A K S** F3

841 AGGCCAGAATGATGGAAAGAATGAGCCTTAAGCACCAAAACAGTGGGAAATGGGCCAAAT 900

Q R Q L W P N M T W R L A K L C R N S W F1

K G N Y G Q I * P G G S P S Y A G T V G F2

**K A I M A K Y D L E A R Q A M Q E Q L A** F3

901 CAAAGGCAATTATGGCCAAATATGACCTGGAGGCTCGCCAAGCTATGCAGGAACAGTTGG 960

P R T K N * H R N S R * P L R V R R R R F1

Q E Q R T D T E T Q G S L * E * G G G G F2

**K N K E L T Q K L K V A S E S E E E E G** F3

961 CCAAGAACAAAGAACTGACACAGAAACTCAAGGTAGCCTCTGAGAGTGAGGAGGAGGAGG 1020

E A Q K W K N S L S L M Q * M K C R * M F1

R R R S G R T P C P * C S E * S A D E C F2

**G A E V E E L L V P D A V N E V Q M N V** F3

1021 GAGGCGCAGAAGTGGAAGAACTCCTTGTCCCTGATGCAGTGAATGAAGTGCAGATGAATG 1080

W T D Q I P G C S G A A P V T P K R L Q F1

G R T K S L D A Q E L H Q * H Q R G C N F2

**D G P N P W M L R S C T S D T K E A A T** F3

1081 TGGACGGACCAAATCCCTGGATGCTCAGGAGCTGCACCAGTGACACCAAAGAGGCTGCAA 1140

P R R R T L S S C Q S L W R T K F L K V F1

R G G G P * A A A R A C G A R S F * K * F2

**E E E D P E Q L P E P V A H E V S E S E** F3

1141 CCGAGGAGGAGGACCCTGAGCAGCTGCCAGAGCCTGTGGCGCACGAAGTTTCTGAAAGTG 1200

R E K K D Q W Q K K T F C * E N L R K G F1

G R R K T S G R R R H F V E R I * G K A F2

**G E E R P V A E E D I L L R E F E E R R** F3

1201 AGGGAGAAGAAAGACCAGTGGCAGAAGAAGACATTTTGTTGAGAGAATTTGAGGAAAGGC 1260

D P L D K D L S S T R M L S Q Q T V K K F1

I P * T K I * A Q P G C * A S R Q S R N F2

**S L R Q R S E L N Q D A E P A D S Q E T** F3

1261 GATCCCTTAGACAAAGATCTGAGCTCAACCAGGATGCTGAGCCAGCAGACAGTCAAGAAA 1320

Q K I L V A R R C C P N * G H C L R N * F1

K R F * * P G G A V R I E G T V S E I E F2

**K D S S S Q E V L S E L R A L S Q K L K** F3

1321 CAAAAGATTCTAGTAGCCAGGAGGTGCTGTCCGAATTGAGGGCACTGTCTCAGAAATTGA 1380

R K T I S P G S K K Q V Q R R L F P R S F1

G R P S V Q E A K S K F R G D C S P G P F2

**E D H Q S R K Q K A S S E A T V P Q V Q** F3

1381 AGGAAGACCATCAGTCCAGGAAGCAAAAAGCAAGTTCAGAGGCGACTGTTCCCCAGGTCC 1440

R E R N L P Q K K R S P C C Y R G Q K E F1

E R G T C P R R R G A P V A T E A R K S F2

**R E E P A P E E E E P L L L Q R P E R V** F3

1441 AGAGAGAGGAACCTGCCCCAGAAGAAGAGGAGCCCCTGTTGCTACAGAGGCCAGAAAGAG 1500

Y R C W K S * K K K N V F K I R S F P D F1

T D A G R A R K R R M F S K * G A F Q T F2

**Q M L E E L E K E E C F Q N K E L S R P** F3

1501 TACAGATGCTGGAAGAGCTAGAAAAAGAAGAATGTTTTCAAAATAAGGAGCTTTCCAGAC 1560

L C * K G I G Q R R P Q I I T L M P L R F1

C V R R A L V R E D P K * S P * C P * G F2

**V L E G H W S E K T P N N H P D A P K E** F3

1561 CTGTGTTAGAAGGGCATTGGTCAGAGAAGACCCCAAATAATCACCCTGATGCCCCTAAGG 1620

R R K R R S K * L T Y R T S * P H S L S F1

E E K E G A N D * P T E P P N H T V S L F2

**K K K K E Q M I D L Q N L L T T Q S L S** F3

1621 AGAAGAAAAAGAAGGAGCAAATGATTGACCTACAGAACCTCCTAACCACACAGTCTCTCT 1680

R * S L W Q C P Q * R C W K M K W R E T F1

G E V F G S A H N R D A G R * S G E K P F2

**V K S L A V P T I E M L E D E V E R N Q** F3

1681 CGGTGAAGTCTTTGGCAGTGCCCACAATAGAGATGCTGGAAGATGAAGTGGAGAGAAACC 1740

K G R * * R K L S L G M M S S E I S * K F1

K A D D K G S F R W G * C H Q R F L E R F2

**R Q M I K E A F A G D D V I R D F L K E** F3

1741 AAAGGCAGATGATAAAGGAAGCTTTCGCTGGGGATGATGTCATCAGAGATTTCTTGAAAG 1800

R R G K L W R P V S Q R T * T * H Y L A F1

E E G S C G G Q * A K G H R P D T T W L F2

**K R E A V E A S K P K D I D L T L P G W** F3

1801 AGAAGAGGGAAGCTGTGGAGGCCAGTAAGCCAAAGGACATAGACCTGACACTACCTGGCT 1860

G A S G V V W A * S P V P R K D A S F S F1

G R V G W C G P K A Q C Q E K T P V S H F2

**G E W G G V G L K P S A K K R R Q F L I** F3

1861 GGGGCGAGTGGGGTGGTGTGGGCCTAAAGCCCAGTGCCAAGAAAAGACGCCAGTTTCTCA 1920

L K P L R V L Q E K I R I C * M * L S M F1

* S P * G S S K K R * E F A K C D Y Q * F2

**K A P E G P P R K D K N L L N V I I N E** F3

1921 TTAAAGCCCCTGAGGGTCCTCCAAGAAAAGATAAGAATTTGCTAAATGTGATTATCAATG 1980

R S A T S T Q Q L I R Y E C F H I H L P F1

E A Q H P R S S S S G T S A S I S I Y P F2

**K R N I H A A A H Q V R V L P Y P F T H** F3

1981 AGAAGCGCAACATCCACGCAGCAGCTCATCAGGTACGAGTGCTTCCATATCCATTTACCC 2040

T I G N L K G P S R P L * D P R G T P R F1

P S A I * K D H P D P Y R I H V E H P E F2

**H R Q F E R T I Q T P I G S T W N T Q R** F3

2041 ACCATCGGCAATTTGAAAGGACCATCCAGACCCCTATAGGATCCACGTGGAACACCCAGA 2100

E L S K S * L L L R S S P S Q A I S L S F1

S F P K A D Y S * G H H Q A R P Y H * A F2

**A F Q K L T T P K V I T K P G H I I K P** F3

2101 GAGCTTTCCAAAAGCTGACTACTCCTAAGGTCATCACCAAGCCAGGCCATATCATTAAGC 2160

P * K Q R M W A T G L P Q G R T S L S Y F1

H K S R G C G L P V F L K V G P L C H T F2

**I K A E D V G Y R S S S R S D L S V I Q** F3

2161 CCATAAAAGCAGAGGATGTGGGCTACCGGTCTTCCTCAAGGTCGGACCTCTCTGTCATAC 2220

R G I Q N G S P H V T K N S * R K T L * F1

E E S K T G H H T S Q K T A E E K L C R F2

**R N P K R V T T R H K K Q L K K N S V D** F3

2221 AGAGGAATCCAAAACGGGTCACCACACGTCACAAAAAACAGCTGAAGAAAAACTCTGTAG 2280

I X F1

L X F2

* F3

2281 ATTGA 2285
